# Peripartum interventions for elevated blood pressure in women with hypertensive disorders of pregnancy: a systematic review and meta-analysis

**DOI:** 10.1016/S3050-5038(25)00183-9

**Published:** 2026-05

**Authors:** Annabelle L Frost, Samuel Krasner, Michelle Li, Prenali D Sattwika, Megha Agarwal, Amelia Shard, Jamie Kitt, Christina Aye, Paul Leeson

**Affiliations:** a Cardiovascular Clinical Research Facility, Division of Cardiovascular Medicine, Radcliffe Department of Medicine, University of Oxford, Oxford, UK; b Department of Internal Medicine, Faculty of Medicine, Public Health, and Nursing, Universitas Gadjah Mada, Yogyakarta, Indonesia; c Women's Centre, Oxford University Hospitals NHS Foundation Trust, Oxford, UK; d Nuffield Department of Women's and Reproductive Health, University of Oxford, Oxford, UK; e National Institute for Health Care and Research, Oxford Biomedical Research Centre, Oxford, UK

## Abstract

**Background:**

Hypertension remains the leading risk factor for cardiovascular disease in women, and those who have a hypertensive disorder of pregnancy are at particular risk. We aimed to determine whether peripartum interventions for hypertension are associated with long-term cardiovascular risk.

**Methods:**

In this systematic review and meta-analysis, we searched six databases—MEDLINE, Embase (via Ovid), the Cochrane Central Register of Controlled Trials, Scopus, Web of Science Core Collection, and the Cumulative Index to Nursing and Allied Health Literature—from database inception to June 18, 2025, for randomised and non-randomised studies that reported on the effect of peripartum interventions (lifestyle, pharmaceutical and nutraceutical, and remote blood pressure management) on cardiovascular outcomes of women with hypertensive disorders of pregnancy. The primary outcome measures of interest were blood pressure, cardiovascular structure and function, and development of cardiovascular disease. We performed random-effects meta-analyses of systolic and diastolic blood pressure outcomes. Other outcomes could not undergo meta-analysis due to heterogeneity of approaches and limited data. Heterogeneity was assessed using the *I*^2^ statistic. Robustness was examined using leave-one-out sensitivity analyses, funnel plots, and Egger's test for small-study effects. GRADE was used to assess the certainty of evidence. The study is registered with PROSPERO, CRD42024581604.

**Findings:**

We identified 11 251 unique records. 34 manuscripts, reporting on 32 unique studies consisting of 3558 women with hypertensive disorders of pregnancy, were included in the systematic review. Most interventions were evaluated up to 2 weeks postpartum; few studies had more than 6 weeks follow-up. 12 of 32 studies were included in the meta-analysis. Pharmaceutical and nutraceutical interventions in the antenatal and postnatal periods did not significantly affect systolic (antenatal period pooled mean difference –3·60 mm Hg [95% CI –14·25 to 7·05], p=0·51, *I*^2^=87·7%; postnatal period –2·69 mm Hg [–5·80 to 0·41], p=0·089, *I*^2^=70·0%) or diastolic (antenatal period –4·58 mm Hg [–11·60 to 2·44], p=0·20, *I*^2^=87·5%; postnatal period –3·30 mm Hg [–7·07 to 0·47], p=0·087, *I*^2^=95·1%) blood pressure up to 1 week postpartum. Remote blood pressure management was associated with significant reductions in both systolic (–8·02 mm Hg [–10·21 to –5·84], p<0·0001, *I*^2^=3·7%) and diastolic (–6·46 mm Hg [–7·85 to –5·07], p<0·0001, *I*^2^=0%) blood pressure within 1 year postpartum. Blood pressure data were insufficient to perform a meta-analysis on lifestyle interventions. Few studies reported on cardiovascular structure and function although improved diastolic function was consistently reported. Clinical outcomes were rarely reported, which led to small sample sizes and low statistical power. Following the GRADE assessment, there was moderate certainty on the effect of antenatal pharmaceutical and nutraceutical therapy and remote blood pressure management on postpartum blood pressure outcomes and limited confidence (low-quality evidence) supporting postnatal pharmaceutical and nutraceutical therapy.

**Interpretation:**

There is a paucity of evidence around peripartum interventions and their effect on modifying cardiovascular risk through blood pressure reduction. There were no high-quality randomised controlled trials reporting long-term outcomes. Future research, with longer follow-up of cardiovascular outcomes or validated surrogate measures of cardiovascular disease, are needed to identify optimal peripartum interventions to reduce risk.

**Funding:**

None.

## Introduction

Hypertension is the leading risk factor for cardiovascular disease and premature mortality in women globally.^1^, ^2^, ^3^, ^4^ Sex differences in blood pressure regulation influence disease progression, with stronger associations found between hypertension and adverse cardiovascular outcomes in women compared with men.^5^ Interventions are typically started later in life in women, when end-organ damage might have already accumulated, which could account for the increased frequency of coronary microvascular dysfunction and heart failure with preserved ejection fraction reported in young women with hypertensive disorders.^2^, ^6^

**Research in context**

**Evidence before this study**

Women with a history of hypertensive disorders in pregnancy are at an increased risk of future cardiovascular disease. Although existing literature predominately supports acute management during pregnancy, there remains a crucial gap in understanding whether interventions around the time of pregnancy influence long-term cardiovascular outcomes. We searched MEDLINE and Embase (via Ovid) for reviews published between Jan 1, 2015, and Aug 16, 2024. Search terms were consistent with the terms used for hypertensive disorders in pregnancy and interventions used in our systematic review (appendix 1 [p 6]). To our knowledge, only two reviews have examined the association between interventions and postpartum cardiovascular health in women with hypertensive diseases in pregnancy. The first study restricted their scope to postnatal interventions, thereby excluding antenatal strategies with postpartum follow-up. This study had a narrower range of outcomes, excluding objective markers of cardiovascular risk, such as imaging or biomarkers. The conclusion based on two trials was a paucity of postpartum randomised controlled trials to reduce cardiovascular risk. A more recent review focused exclusively on nutrition-based interventions and showed non-significant changes in blood pressure postpartum. The first review included randomised controlled trials in women less than 10 years postpartum with a history of hypertensive disorders in pregnancy, interventions aimed at reducing long-term cardiovascular risk and outcomes of cardiovascular disease events, risk markers of cardiovascular disease, and markers of compliance to assess feasibility. They excluded women with a primary cause for cardiovascular disease as well as trials in women with other conditions such as gestational diabetes or high BMI. There was no report on quality of evidence included but studies with poor quality of evidence were not included. The second review also included women less than 10 years postpartum with hypertensive disorders in pregnancy. It included all types of studies with a nutrition-focused intervention and with outcomes of markers of cardiovascular risk. The quality of evidence was moderate, with only one experimental study having a low risk of bias.

**Added value of this study**

This systematic review and meta-analysis expands on previous work by evaluating a broader range of antenatal and postnatal interventions and their effects on postpartum cardiovascular health in women with hypertensive disorders of pregnancy. In doing so, we highlight early signals that some pregnancy-related interventions might influence long-term cardiovascular outcomes and identify the need for more systematic, long-term investigations.

**Implications of all the available evidence**

Our study suggests that remote blood pressure monitoring might improve cardiovascular risk, as indicated by lower blood pressure at 1-year postpartum. However, short-term follow-up limits understanding of an intervention and its effect on long-term cardiovascular outcomes. Without robust evidence on the effect of antenatal and postnatal interventions on future cardiovascular risk, progress in developing targeted strategies for this population remains limited. Research into the management of hypertensive disorders in pregnancy should include sufficient follow-up of cardiovascular outcomes or validated surrogate markers of cardiovascular disease to accurately assess the medium-term to long-term effects of interventions.

Hypertensive disorders of pregnancy are a sex-specific risk factor that can be used to identify women earlier in life as having an increased risk of atherosclerosis, stroke, and heart failure. A fifth of women with pre-eclampsia develop hypertension within 15 years of pregnancy.^7^, ^8^ These women have earlier vascular stiffness, systolic hypertension, cardiac hypertrophy, and microvascular damage and display end-organ disease at lower blood pressures.^9^, ^10^, ^11^, ^12^, ^13^ Despite the recognised and burdening risk of hypertensive disorders in pregnancy, there are discrepancies in management strategies internationally, particularly with thresholds for the commencement of pharmaceutical and non-pharmacological interventions.^14^ The 2025 European Society of Cardiology guidelines now recommend a blood pressure threshold of ≤140/90 mm Hg, supporting tighter blood pressure control during and after pregnancy.^15^ These sex-specific changes highlight pregnancy and the postpartum period as potentially unique junctions in the life of a woman, which could change their risk factor trajectory and prevent early onset cardiovascular disease.^16^

The vascular basis of pregnancy hypertension means there have been multiple trials of cardiovascular, pharmacological, and lifestyle interventions to prevent or reduce the effects of pre-eclampsia. Trial design traditionally focuses on immediate risk reduction, such as blood pressure change during pregnancy. However, cardiovascular prevention interventions that modify pregnancy risk could also affect risk exposure after pregnancy either by reducing organ damage during pregnancy or by earlier identification of risk factors that require management. We aimed to investigate the effects of prepartum and postpartum cardiovascular prevention interventions on long-term cardiovascular risk.

## Methods

### Search strategy and selection criteria

The search terms for this systematic review and meta-analysis were developed by combining key terms and medical language related to the spectrum of hypertensive disorders of pregnancy, interventions, and surrogate markers of the cardiovascular system. Full search terms are described in appendix 1 (pp 6–7). We searched MEDLINE and Embase (via Ovid), Cochrane Central Register of Controlled Trials, Scopus, Web of Science core collection, and the Cumulative Index to Nursing and Allied Health Literature. One reviewer (ALF) searched for articles published from database inception to June 18, 2025, without any language or study type restrictions.

Studies assessing interventions performed in women diagnosed with gestational hypertension, pre-eclampsia, and superimposed pre-eclampsia in pregnancy were included in the systematic review. RCTs were included in the meta-analyses because of the relevant data and ability to perform the analyses. The exposed group comprised women diagnosed with hypertensive disorders of pregnancy who received a peripartum intervention; participants in the control group received no intervention. Interventions included pharmaceutical and nutraceutical therapy, lifestyle, and remote blood pressure management. For the purpose of this systematic review, a nutraceutical is defined as a supplement, including amino acids and probiotics. Explanations of terminology and examples of categorisation of interventions are detailed in appendix 1 (p 11). The primary outcomes included phenotypic and surrogate measures of cardiovascular risk, such as blood pressure, cardiovascular structure and function, and development of cardiovascular disease.

Following database searches, records were input into the Evidence Review Accelerator (TERA) for deduplication.^17^ All remaining unique abstracts were independently screened by at least two authors (ALF, SK, PDS, ML, AS, and MA) using Rayyan software.^18^ Abstracts fulfilling the inclusion criteria were selected for full-text review. The full inclusion criteria are detailed in appendix 1 (pp 7–8). Randomised and non-randomised studies were included. Studies were excluded if there was no record of phenotypic or surrogate markers of cardiovascular risk, or if the data could not be extracted from the study groups. Selection discrepancies following a full-text review were resolved through discussion with at least two authors (ALF, SK, PDS, ML, AS, and MA). The full list of studies excluded during full-text screening is available in appendix 1 (pp 12–31).

Data were extracted using a standardised form (appendix 2) by three reviewers (ALF and SK extracted data for all studies, which was adjudicated by ML). Any disagreement was resolved by discussion. Full details of the extracted data are described in appendix 1 (p 8).

An institutional board or ethics committee review was not required. The systematic review was done in accordance with the Cochrane Handbook for Systematic Reviews of Interventions^19^ and reported in line with PRISMA guidelines (appendix 1 [pp 2–5]).^20^ The review protocol was registered with PROSPERO (CRD42024581604).

### Data analysis

A risk of bias assessment was independently done for the included randomised controlled trials (RCTs) by two reviewers (ALF and SK) using the Cochrane Risk-of-Bias tool. The quality of trials included was assessed using GRADE. Details of the method and results are described in appendix 1 (RoB 2 [pp 9–10]; GRADE assessments [pp 32–33]).

Findings were grouped according to intervention type (pharmaceutical and nutraceutical therapy, lifestyle, and remote blood pressure management) and timing of intervention (either antenatal or postnatal). Meta-analysis were done if two or more studies evaluated the same intervention category at similar timepoints (defined as within 1 week postpartum, 1–6 weeks postpartum, or 6 weeks to 1 year postpartum, reflecting the immediate to short-term assessment of cardiovascular risk) and reported both systolic and diastolic blood pressure outcomes. Meta-analyses were done using RStudio (version 2025.05.0+496) with the meta and metafor packages.^21^, ^22^ Pooled effect estimates were calculated using a random-effects model to account for between-study heterogeneity. Forest plots were generated to visually represent effect sizes and 95% CIs. For each study, the mean difference (MD) and corresponding SE were used to calculate a test statistic (Z=MD/SE). Two-sided p values were derived from the standard normal distribution. Heterogeneity was assessed using the *I*^2^ statistic. Publication bias was evaluated through visual inspection of funnel plots and Egger's test. Subgroup and leave-one-out sensitivity analyses were done to explore sources of heterogeneity and assess the robustness of the findings.

### Role of the funding source

There was no funding source for this study.

## Results

**Figure 1:**
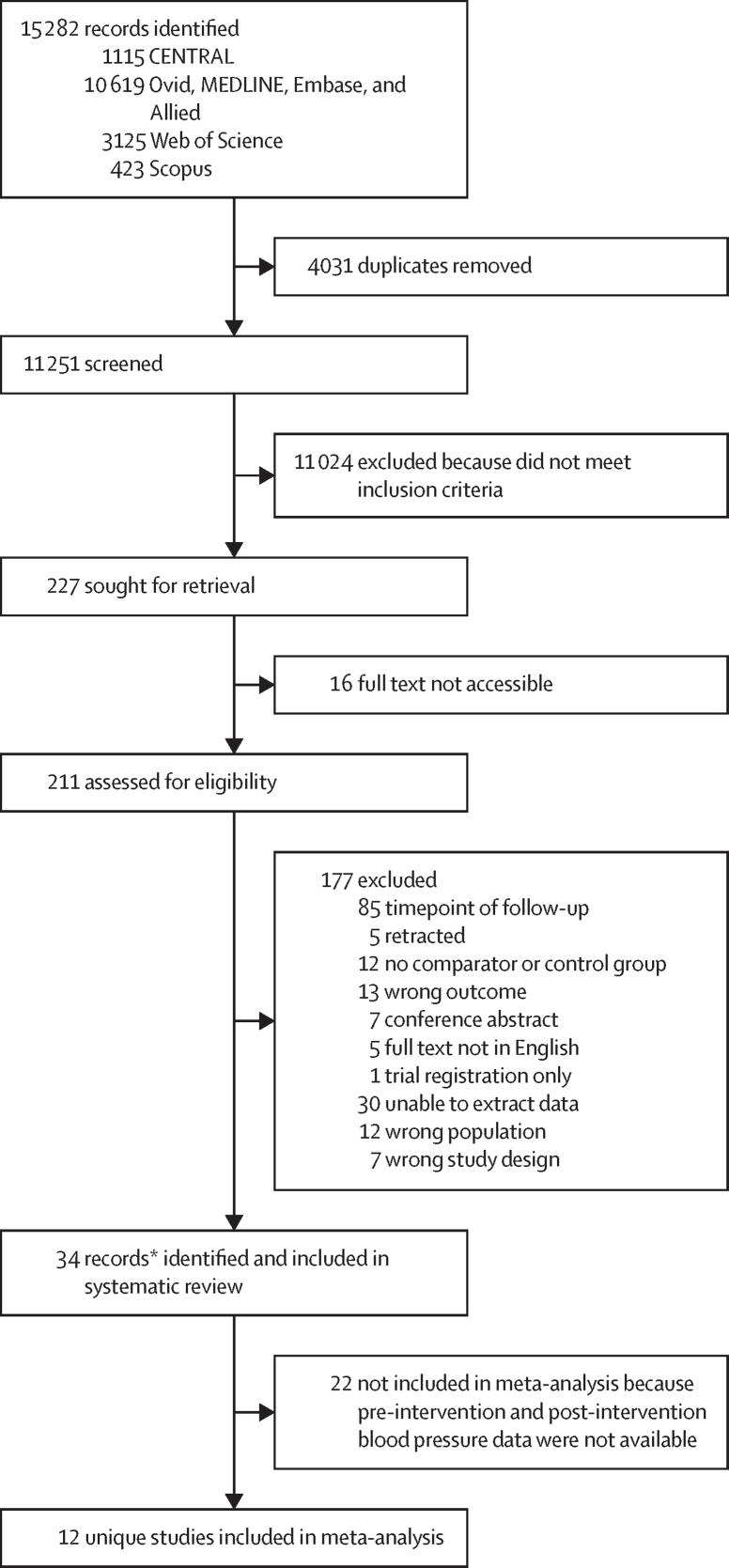
Study selection CENTRAL=Cochrane Central Register of Controlled Trials. *Reported data from 32 unique clinical trials or studies.

The search strategy identified 11 251 unique references, 34 of which were included in the systematic review.^23^, ^24^, ^25^, ^26^, ^27^, ^28^, ^29^, ^30^, ^31^, ^32^, ^33^, ^34^, ^35^, ^36^, ^37^, ^38^, ^39^, ^40^, ^41^, ^42^, ^43^, ^44^, ^45^, ^46^, ^47^, ^48^, ^49^, ^50^, ^51^, ^52^, ^53^, ^54^, ^55^, ^56^ These 34 publications reported data from 32 unique studies, which included data from 3558 women with hypertensive disorders of pregnancy and reported outcome data at multiple timepoints. 12 of these studies were included in the meta-analysis (figure 1).^25^, ^29^, ^31^, ^32^, ^33^, ^42^, ^44^, ^45^, ^50^, ^51^, ^53^, ^55^ One non-RCT (a case-control study)^49^ was included due to documented outcomes of cardiovascular risk following an intervention as well as follow-up in the correct population. The list of included studies and data describing study design and setting, type of hypertensive disorder of pregnancy, timing of intervention, as well as type and duration are included in table 1 and appendix 1 (pp 34–42).

**Table 1:** Summary of included studies and their characteristics

	**Country and setting**	**Study design**	**Population**		**Intervention**					**Outcomes**			
			Number of patients with hypertensive disorder of pregnancy	Hypertensive disorder of pregnancy type	Intervention start point	Intervention type	Intervention description	Intervention duration	Control	Timepoint of measurements after intervention*	Blood pressure†	Development of cardiovascular disease‡	Cardiovascular structure and function§
Adair et al (2009)^23^	USA; hospital	Double-blind RCT	26 (13 intervention, 13 control)	Pre-eclampsia	Postpartum	Pharmaceutical and nutraceutical therapy	Digoxin-binding Fab fragment immunoglobulin	Within 15 min of clamping of umbilical cord after birth; administered as an intravenous bolus over 10 min	Placebo (saline)	24 h	Yes	No	No
Aderibigbe et al (2023)^24^	USA; hospital	Open-label RCT	256 (128 intervention, 128 control)	Gestational or chronic hypertension	Postpartum	Pharmaceutical and nutraceutical therapy	Tight control of blood pressure (<140/90 mm Hg)	Beginning immediately after birth; ending not specified	Liberal control of blood pressure (<150/95 mm Hg)	Not specified¶	Yes	No	No
Arias-Hernández et al (2020)^25^	Mexico; hospital	Single-blind RCT	42 (21 intervention, 21 control)	Pre-eclampsia	Postpartum	Pharmaceutical and nutraceutical therapy	Diltiazem	2 days; beginning within 1 day after birth	Nifedipine (10 mg)	2 days	Yes	No	No
Bartal et al (2023)^26^	USA; hospital	Open-label RCT	58 (30 intervention, 28 control)	Gestational or chronic hypertension	Postpartum	Pharmaceutical and nutraceutical therapy	Combined hydrochlorothiazide and lisinopril	Beginning within 3 days of giving birth until first postpartum visit 7–10 days after birth	Nifedipine (30 mg)	10 days	Yes	Yes	No
Barton et al (1990)^27^	USA; hospital	Double-blind RCT	31 (16 intervention, 15 control)	Severe pre-eclampsia	Postpartum	Pharmaceutical and nutraceutical therapy	Nifedipine	2 days; beginning immediately after birth	Placebo	2 days	No	No	No
Blue et al (2018)^28^	USA; hospital	Double-blind RCT	93 (46 intervention, 47 control)	Pre-eclampsia, chronic hypertension, or HELLP syndrome	Postpartum	Pharmaceutical and nutraceutical therapy	Ibuprofen	Beginning within 6 h after birth, until discharge (minimum of 3 days)	Paracetamol (650 mg)	3 days	Yes	No	No
Cairns et al (2018)^29^	UK; home	Open-label RCT	82 (40 intervention, 42 control)	Gestational hypertension or pre-eclampsia	Postpartum	Remote blood pressure management	Daily blood pressure self-monitoring	6 months; beginning after discharge	Usual care	6 weeks and 6 months	Yes	No	No
Chappell et al (2022)^30^	UK; home	Open-label RCT	377 (187 intervention, 190 control)	Gestational or chronic hypertension	Second or third trimester	Remote blood pressure management	Daily blood pressure self-monitoring	Beginning at recruitment until birth	Usual care	8 weeks	No	No	No
Cluver et al (2021)^31^	South Africa; hospital	Double-blind RCT	179 (89 intervention, 90 control)	Pre-eclampsia	Second or third trimester	Pharmaceutical and nutraceutical therapy	Metformin	Beginning at recruitment until birth	Placebo	Day of delivery	Yes	Yes	No
Cursino et al (2025)^32^	Brazil; hospital	Triple-blind RCT	118 (58 intervention, 60 control)	Pre-eclampsia	Postpartum	Pharmaceutical and nutraceutical therapy	Furosemide	5 days; beginning 1 day after birth	Placebo	5 days	Yes	No	No
Dabaghi et al (2019)^33^	Iran; hospital	Single-blind RCT	90 (45 intervention, 45 control)	Pre-eclampsia or HELLP syndrome	Postpartum	Pharmaceutical and nutraceutical therapy	Furosemide	5 days; beginning immediately after birth	No medication	3 days	Yes	Yes	No
Elhassan et al (2002)^34^	Sudan; hospital	Open-label RCT	70 (34 intervention, 36 control)	Pre-eclampsia	Third trimester	Pharmaceutical and nutraceutical therapy	Methyldopa	Beginning at recruitment, until giving birth	No medication	6 weeks	Yes	No	No
Elzayyat and Yacoub (2014)^35^	Egypt; hospital	Single-blind RCT	50 (25 intervention, 25 control)	Pre-eclampsia	Third trimester	Pharmaceutical and nutraceutical therapy	Magnesium sulphate	Beginning 30 min pre-birth until 1 day after birth	No medication	6 h	No	No	Yes
Hashemi et al (2016)^36^	Iran; hospital	Triple-blind RCT	75 (38 intervention, 37 control)	Pre-eclampsia	Second or third trimester	Pharmaceutical and nutraceutical therapy	Aspirin	Beginning at recruitment, until 2 months after birth	Placebo	2 months	No	No	Yes
Hauspurg et al (2023)^37^	USA; home	Open-label RCT	129 (81 intervention, 48 control)	Gestational hypertension or pre-eclampsia	Postpartum	Lifestyle	Daily blood pressure self-monitoring with or without electric scale with self-help website access	6 months; beginning within 6 months of giving birth	Self-help website access	6 months	Yes	No	No
Hladunewich et al (2006)^38^	USA; hospital	Double-blind RCT	39 (19 intervention, 20 control)	Pre-eclampsia	Third trimester	Pharmaceutical and nutraceutical therapy	L-arginine	Beginning within 1 day before delivery or immediately after birth until 10 days post-delivery	Placebo	10 days	Yes	No	No
Hutchesson et al (2020)^39^	Australia; home	Single-blind RCT	31 (16 intervention, 15 control)	Pre-eclampsia	Postpartum	Lifestyle	Access to a lifestyle behaviour intervention	3 months	Email with links to National Heart Foundation of Australia website	3 months	No	No	No
Isler et al (2003)^40^	USA; hospital	Double-blind RCT	36 (18 intervention, 18 control)	HELLP syndrome	Postpartum	Pharmaceutical and nutraceutical therapy	Dexamethasone	Beginning at recruitment, until criteria for discontinuation of the medication were fulfilled	Betamethasone (12 mg per day)	Not specified¶	Yes	No	No
Kitt et al (2021)^41^	UK; home	Open-label RCT	61 (30 intervention, 31 control)	Gestational hypertension or pre-eclampsia	Postpartum	Remote blood pressure management	Daily blood pressure self-monitoring	6 months; beginning after discharge	Usual care	4 years	Yes	No	No
Kitt et al (2023)^42^	UK; home	Open-label RCT	200 (105 intervention, 95 control)	Gestational hypertension or pre-eclampsia	Postpartum	Remote blood pressure management	Daily blood pressure self-monitoring	Beginning within 1–6 days after birth; ending not specified	Usual care	6 weeks and 9 months	Yes	No	No
Kitt et al (2024)^43^	UK; home	Open-label RCT	187 (101 intervention, 86 control)	Gestational hypertension or pre-eclampsia	Postpartum	Remote blood pressure management	Daily blood pressure self-monitoring	Beginning within 1–6 days after birth; ending not specified	Usual care	9 months	No	No	Yes
Movaghar et al (2024)^44^	Iran; hospital or at home	Triple-blind RCT	128 (64 intervention, 64 control)	Pre-eclampsia	Second or third trimester	Pharmaceutical and nutraceutical therapy	Synbiotic capsule containing prebiotics and probiotics	Beginning at recruitment, until giving birth	Placebo	Day of delivery	Yes	Yes	No
Noronha Neto et al (2017)^45^	Brazil; hospital	Triple-blind RCT	88 (43 intervention, 45 control)	Gestational hypertension or pre-eclampsia	Postpartum	Pharmaceutical and nutraceutical therapy	Clonidine	4 days; beginning at recruitment after birth	Captopril (25 mg)	4 days	Yes	Yes	No
Ormesher et al (2020)^46^	UK; hospital	Double-blind RCT	40 (19 intervention, 21 control)	Pre-eclampsia	Postpartum	Pharmaceutical and nutraceutical therapy	Enalapril	6 months; beginning within 3 days after birth	Placebo	6 weeks and 6 months	Yes	No	Yes
Paidas et al (2020)^47^	USA; hospital	Double-blind RCT	120 (62 intervention, 58 control)	Pre-eclampsia and superimposed pre-eclampsia	Second or third trimester	Pharmaceutical and nutraceutical therapy	Recombinant antithrombin therapy	Beginning at recruitment, until giving birth	Placebo	6 weeks	No	Yes	No
Perdigao et al (2021)^48^	USA; hospital	Double-blind RCT	331 (168 intervention, 163 control)	Pre-eclampsia and superimposed pre-eclampsia	Postpartum	Pharmaceutical and nutraceutical therapy	Furosemide	5 days; beginning within 1 day after birth	Placebo	7 days	No	Yes	No
Pihelgas et al (2024)^49^	Canada; home	Case-control study	157 (94 intervention, 63 control)	Pre-eclampsia	Postpartum	Lifestyle	Referred to postpartum pre-eclampsia clinic	1 year postpartum	Missed or declined referral to postpartum pre-eclampsia clinic	1–10 years	No	Yes	No
Siamansoori et al (2020)^50^	Iran; hospital	Double-blind RCT	100 (50 intervention, 50 control)	Pre-eclampsia	Postpartum	Pharmaceutical and nutraceutical therapy	Furosemide	Not defined	Methyldopa (750 mg per day)	7 days	Yes	No	No
Suganya et al (2024)^51^	India; hospital	Single-blind RCT	120 (60 intervention, 60 control)	Pre-eclampsia	Postpartum	Pharmaceutical and nutraceutical therapy	Furosemide and labetalol	5 days; beginning immediately after birth	Labetalol (dose not specified)	5 days	Yes	Yes	No
Trapani et al (2016)^52^	Brazil; hospital	Double-blind RCT	93 (47 intervention, 46 control)	Pre-eclampsia	Second or third trimester	Pharmaceutical and nutraceutical therapy	Sildenafil citrate	Beginning at recruitment, until birth	Placebo	Day of delivery	No	Yes	No
Vigil-De Gracia and García-Cáceres (1997)^53^	Mexico; hospital	RCT	34 (17 intervention, 17 control)	HELLP syndrome	Postpartum	Pharmaceutical and nutraceutical therapy	Dexamethasone	1 day; beginning immediately after birth	No medication	3 days	Yes	No	No
Viteri et al (2018)^54^	USA; hospital or at home	Double-blind RCT	118 (59 intervention, 59 control)	Pre-eclampsia	Postpartum	Pharmaceutical and nutraceutical therapy	Torsemide	5 days; beginning within 1 day after birth	Placebo	5 days	No	No	No
Wang et al (2018)^55^	China; hospital	Double-blind RCT	143 (71 intervention, 72 control)	Pre-eclampsia	Antenatal (specific trimester not specified)	Pharmaceutical and nutraceutical therapy	Nifedipine	4 h; beginning at recruitment until target blood pressure reached	Labetalol in escalating doses of 20 mg, 40 mg, 80 mg, 80 mg, and 80 mg every 20 min until target blood pressure reached	4 h post -intervention	No	Yes	No
Wang et al (2024)^56^	USA; home	Open-label RCT	104 (53 intervention, 51 control)	Gestational hypertension or pre-eclampsia	Postpartum	Lifestyle	Use of neonatal sleep aid	4 months; beginning after discharge	Usual care (safe sleep education)	6 weeks	Yes	No	No

RCT=randomised control trial. HELLP=haemolysis, elevated liver enzymes, and low platelets.

* Time measured from day of giving birth.

† Home and office blood pressure measurements, including when only mean arterial pressure was reported.

‡ Includes conditions such as heart failure, myocardial infarction, angina, stroke, and renal failure; reported cardiovascular events are detailed in table 2.

§ Includes assessment of left ventricular and right ventricular mass and markers of endothelial dysfunction, assessed by multimodality imaging, such as echocardiography, cardiac MRI, and flow-mediated dilation; also includes serum biomarkers, such as NT-proBNP, cardiac troponin, sFlt-1, and placental growth factor, as well as arteriography, such as pulse wave velocity and the augmentation index.

¶ Primary endpoint timing was variable and not clearly described.

Pharmaceutical and nutraceutical interventions were had a moderate quality of evidence when initiated antenatally and a low quality of evidence when initiated postnatally. One RCT^25^ was downgraded by one level in risk of bias due to a lack of masking, and deviation from intended interventions was found in two studies.^25^, ^56^ Remote blood pressure management postnatally had moderate quality evidence, which was downgraded for sample size.

The distribution of study follow-up periods and intervention types across the included studies reporting blood pressure outcomes is shown in appendix 1 (p 44). Most studies reported short-term outcomes, 15 (47%) studies contributing data up to 1 week postpartum. Fewer studies with longer follow-up periods were found, with one (3%) study with up to 2 weeks,^26^ six (19%) with up to 6 weeks,^29^, ^34^, ^42^, ^46^, ^47^, ^56^ six (19%) with up to 6 months,^29^, ^30^, ^36^, ^39^, ^46^, ^37^ two (6%) with up to 1 year,^42^, ^43^ one (3%) with up to 5 years,^41^ and one (3%) with up to 10 years.^49^ Pharmaceutical and nutraceutical interventions (25 [78%] studies) were the most common among studies with short-term follow-up, particularly up to 2 weeks postpartum. Remote blood pressure management and lifestyle interventions were more likely to report longer follow-up periods, particularly at 6 months postpartum, but also up to 10 years after delivery.

We evaluated the effects of pharmaceutical and nutraceutical therapy and remote blood pressure management on postnatal systolic and diastolic blood pressure using meta-analyses. There were insufficient blood pressure data to perform a meta-analysis on lifestyle interventions.

**Figure 2:**
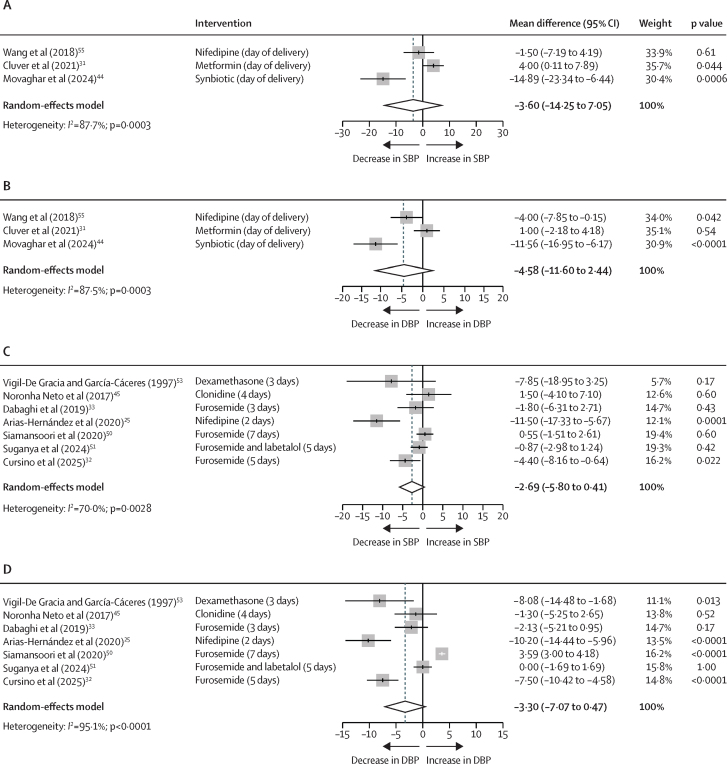
Forest plots illustrating the effects of pharmaceutical and nutraceutical interventions on postnatal SBP and DBP (mm Hg), stratified by the timing of the intervention The effects of antenatal pharmaceutical and nutraceutical interventions (administered before birth) on postnatal SBP (A) and DBP (B). The effects of postnatal pharmaceutical and nutraceutical interventions (administered after birth) on postnatal SBP (C) and DBP (D). In all studies, blood pressure outcomes were measured postnatally, regardless of when the intervention occurred. The exact postnatal timepoint of blood pressure measurement varied between study and is specified in brackets, with the pharmaceutical and nutraceutical intervention used. These timepoints range from the day of delivery to 1-week postpartum. Articles included.^25^, ^31^, ^32^, ^33^, ^44^, ^45^, ^50^, ^51^, ^53^, ^55^ DBP=diastolic blood pressure. SBP=systolic blood pressure.

Antenatal pharmaceutical and nutraceutical interventions (figure 2) did not affect systolic blood pressure within 1 week after birth, with a pooled mean difference of –3·60 mm Hg (95% CI –14·25 to 7·05; p=0·51; *I*^2^=87·7%). Equally, antenatal pharmaceutical and nutraceutical interventions (figure 2) did not affect diastolic blood pressure, with a pooled mean difference of –4·58 mm Hg (–11·60 to 2·44; p=0·20; *I*^2^=87·5%).

Postnatal pharmaceutical and nutraceutical interventions (figure 2) did not affect systolic blood pressure, with a pooled mean difference of –2·69 mm Hg (95% CI –5·80 to 0·41; p=0·089, *I*^2^=70·0%). These studies initiated medications after delivery with an observation period ranging from 2 days to 7 days after birth. Postnatal pharmaceutical and nutraceutical interventions (figure 2) showed no effect on diastolic blood pressure within the same time frame, with a pooled mean difference of –3·30 mm Hg (–7·07 to 0·47; p=0·087; *I*^2^=95·1%). To identify potential sources for the high heterogeneity, subgroup analyses were performed but no single study contributed disproportionately (appendix 1 [pp 45–46]).

**Figure 3:**
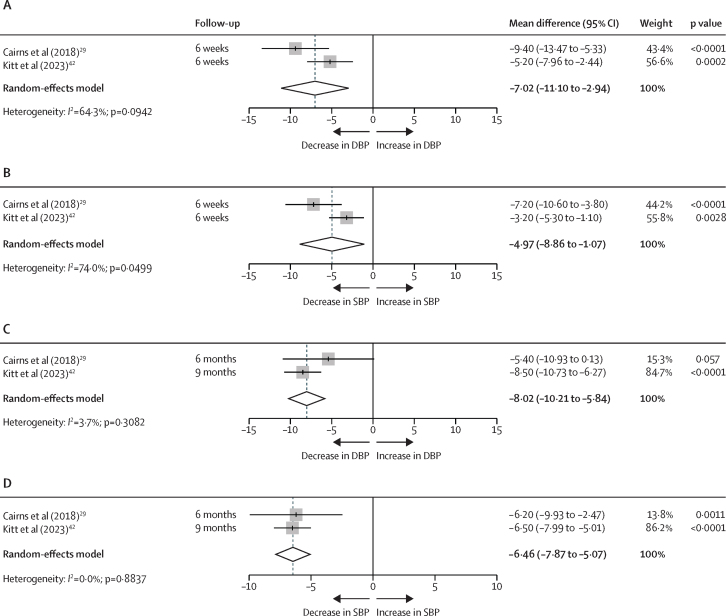
Forest plots illustrating the effects of postnatal remote blood pressure management interventions on postnatal SBP and DBP (mm Hg) The effect of postnatal remote blood pressure management on DBP (A) and SBP (B) 1–6 weeks postpartum. The effect of postnatal remote blood pressure management on DBP (C) and SBP (D) between 6 weeks and 1 year postpartum. Included studies evaluated the use of remote blood pressure management as an intervention, which was implemented after birth. The time of follow-up for each study is specified in brackets. Articles included.^29^, ^42^ DBP=diastolic blood pressure. SBP=systolic blood pressure.

Postnatal remote blood pressure management was associated with significantly lower blood pressure across both early (1–6 weeks postpartum; figure 3A, B) and later (6 weeks to 1 year postpartum; figure 3C, D) postpartum assessments. When measured within 1–6 weeks postpartum, pooled analyses showed a change in systolic blood pressure of –7·02 mm Hg (95% CI –11·10 to –2·94; p=0·0007; *I*^2^=64·3%) and diastolic blood pressure of –4·97 mm Hg (–8·86 to –1·07; p=0·012; *I*^2^=74·0%). At long-term follow-up (6 weeks to 1 year postpartum), interventions continued to show significant benefit, with greater reductions observed in systolic blood pressure (mean difference –8·02 mm Hg [–10·21 to –5·84]; p<0·0001; *I*^2^=3·7%) and diastolic blood pressure (–6·46 mm Hg [–7·85 to –5·07]; p<0·0001; *I*^2^=0·0%).

Visual inspection of funnel plots suggested no major asymmetry for studies reporting systolic blood pressure, whereas there was some asymmetry in the funnel plots for diastolic blood pressure, particularly in the reporting of smaller studies (appendix 1 [p 47]). Egger's linear regression test supported these observations, showing no statistically significant evidence of small-study effects for systolic blood pressure (p=0·14), but evidence of asymmetry for diastolic blood pressure (p<0·0001).

Four studies reported outcomes on cardiovascular structure and function.^35^, ^36^, ^43^, ^46^ Across these studies, measurements included transthoracic echocardiography and cardiac MRI, vascular function indices (eg, brachial artery flow-mediated dilation), indices of arterial stiffness (eg, pulse wave velocity and augmentation index), and circulating biomarkers. Following a 6-month postnatal course of enalapril, better diastolic function and more favourable left ventricular remodelling were observed, with no between-group difference in total vascular resistance or systolic function by global longitudinal strain. Additionally, surrogate biomarkers, such as hs-cTnT and NT-proBNP, were within the normal range in both groups and angiogenic biomarkers did not differ by allocation.^46^ Remote blood pressure management was also associated with a greater reduction in relative wall thickness on echocardiography, lower left ventricular mass and volumes on cardiac MRI, increased left ventricular systolic function, improved global longitudinal strain, and improved diastolic function.^43^ Improved endothelial function following low-dose aspirin was seen with flow-mediated dilation increasing from 9·61% (SD 5·58) at baseline to 13·65% (SD 7·91), whereas the placebo group showed no significant change.^36^ Preoperative magnesium sulphate in women with moderate pre-eclampsia did not reduce evidence of differential myocardial injury using cardiac troponin as a biomarker.^35^

Clinical outcome data were reported across cardiovascular, cerebrovascular, renal, endocrine, and pregnancy-related endpoints (table 2). Overall, 28 unique comparisons from ten (31%) of 32 studies were included. Most outcomes were rare and sample sizes were generally small, restricting statistical power. The timepoint of reported outcomes ranged from day of delivery to up to 10 years postpartum. Nine of ten studies reported outcome data within the first 2 weeks postpartum; one study reported outcomes up to 10 years postpartum.^49^ Development of cardiovascular disease was reported by interventions trialling the use of antihypertensives, diuretics, anticoagulants, sildenafil, and metformin, as well as the effect on lowering the threshold of use of antihypertensives (table 2).

**Table 2:** Maternal outcomes extracted from included trials, grouped by clinical category

	**Study**	**Follow-up timepoint**	**Intervention**			**Control**		
			Group	Events	Number of women	Group	Events	Number of women
**Cerebrovascular**								
Posterior reversible encephalopathy syndrome	Suganya et al (2024)^51^	5 days	Furosemide and labetalol	1	60	Labetalol	1	60
Stroke	Paidas et al (2020)^47^	6 weeks	Recombinant antithrombin	0	62	Placebo	0	58
Stroke	Pihelgas et al (2024)^49^	1–10 years	Attended PPPC	0	91	Usual care	1	59
Amarousis fugax	Suganya et al (2024)^51^	5 days	Furosemide and labetalol	0	60	Labetalol	1	60
**Cardiovascular**								
Cardiomyopathy	Bartal et al (2023)^57^	10 days	Lisinopril and hydrochlorothiazide	1	31	Nifedipine	0	36
Pulmonary oedema	Bartal et al (2023)^57^	10 days	Lisinopril and hydrochlorothiazide	1	31	Nifedipine	0	36
Pulmonary oedema	Cluver et al (2021)^31^	Day of birth	Metformin	4	89	Placebo	2	90
Pulmonary oedema	Noronha Neto et al (2017)^45^	4 days	Clonidine	1	43	Captopril	0	45
Pulmonary oedema	Paidas et al (2020)^47^	6 weeks	Recombinant antithrombin	2	62	Placebo	3	58
Pulmonary oedema	Perdigao et al (2021)^48^	7 days	Furosemide	3	192	Placebo	1	192
Pulmonary oedema	Suganya et al (2024)^51^	5 days	Furosemide and labetalol	0	60	Labetalol	5	60
Cardiac failure	Trapani at al (2016)^52^	Day of birth	Sildenafil	0	50	Placebo	1	50
Cardiac failure	Cluver et al (2021)^31^	Day of birth	Metformin	0	89	Placebo	1	90
Cardiac failure	Wang et al (2018)^55^	4 h post intervention	Nifedipine	2	71	Labetalol	1	72
Cardiac failure	Pihelgas et al (2024)^49^	1–10 years	Attended PPPC	0	92	Usual care	3	3
Coronary artery disease	Pihelgas et al (2024)^49^	1–10 years	Attended PPPC	0	92	Usual care	0	60
Hypertension	Pihelgas et al (2024)^49^	1–10 years	Attended PPPC	7	93	Usual care	14	62
Severe hypertension	Trapani et al (2016)^52^	Day of birth	Sildenafil	11	50	Placebo	15	50
On antihypertensive after 14 days postpartum	Aderibigbe et al (2023)^24^	Not specified*	Tight blood pressure control	44	128	Liberal control	32	128
Persistent hypertension at 7 days postpartum	Perdigao et al (2021)^48^	7 days	Furosemide	10	192	Placebo	23	192
High cholesterol	Pihelgas et al (2024)^49^	1–10 years	Attended PPPC	4	86	Usual care	2	58
**Renal**								
Acute kidney injury	Aderibigbe et al (2023)^24^	Not specified*	Tight blood pressure control	2	128	Liberal control	3	128
Acute renal failure	Wang et al (2018)^55^	4 h post intervention	Nifedipine	3	71	Labetalol	4	72
Chronic kidney disease	Pihelgas et al (2024)^49^	1–10 years	Attended PPPC	0	90	Usual care	1	60
**Pregnancy**								
Eclampsia	Trapani et al (2016)^52^	Day of birth	Sildenafil	2	50	Placebo	3	50
**Endocrine**								
Diabetes	Pihelgas et al (2024)^49^	1–10 years	Attended PPPC	1	92	Usual care	4	60
**Other**								
Serous retinal detachment	Suganya et al (2024)^51^	5 days	Furosemide and labetalol	0	60	Labetalol	2	60

For each outcome, the intervention and control groups are listed alongside the number of events and the number of participants analysed. Outcomes include cardiovascular, cerebrovascular, renal, endocrine, and obstetric complications. Data reflect events occurring during the reported follow-up period, as defined by each study. The follow-up period ranges from day of delivery to up to 10 years postpartum. Due to heterogeneity in study design, population, and outcome definitions, results are presented descriptively without meta-analysis. PPPC=postpartum pre-eclampsia clinic.

* Primary endpoint timing was variable and not clearly described.

Cerebrovascular events were infrequently reported. Two studies reported isolated cases of stroke, amaurosis fugax, and posterior reversible encephalopathy syndrome, with no clear differences between intervention and control groups at 5 days and 10 years.^49^, ^51^

Cardiovascular outcomes were the most frequently reported. Pulmonary oedema was assessed in six (60%) of ten studies. A trial of furosemide and labetalol expectedly reduced pulmonary oedema rates when compared with labetalol alone,^51^ whereas metformin increased the number of events at the time of giving birth.^31^ Heart failure and cardiomyopathy were also evaluated, with no consistent effect of intervention across five studies.^49^ One study reported a significant reduction in persistent hypertension at 7 days postpartum was observed with furosemide compared with placebo.^48^

Endocrine and renal outcomes were sparsely reported. After 10 years, the incidence of diabetes was lower in the intervention group in one study,^49^ and no events of chronic kidney disease were reported in any intervention group across all ten studies. Rates of acute kidney injury and acute renal failure were similar across groups, both in the immediate postpartum period and after long-term follow-up.

Other maternal complications included serous retinal detachment, which occurred more frequently in the control group,^51^ and eclampsia, which showed no consistent difference between groups.^52^

## Discussion

Our systematic review aimed to evaluate whether peripartum interventions in women with hypertensive disorders of pregnancy might affect long-term cardiovascular outcomes. Our findings suggest a role for remote blood pressure management delivered postnatally. There is a paucity of high-quality RCTs with few studies reporting outcomes beyond 2 weeks postpartum.

The meta-analysis of blood pressure outcomes indicated that the most consistent and clinically significant improvements in both systolic and diastolic blood pressure were achieved through postnatal remote blood pressure management. These interventions were the only category to significantly reduce systolic blood pressure in women with hypertensive disorders of pregnancy between giving birth and 1 year postpartum. This reduction is clinically relevant because a 5 mm Hg difference in blood pressure in later life equates to a 10% relative risk reduction for vascular events.^58^ Remote blood pressure management interventions combine patient autonomy, accurate blood pressure readings, and timely management by physicians using pharmaceuticals. It is this combination that could contribute to the superior effect on blood pressure compared with interventions using one element.^24^, ^42^, ^37^ The contribution of patient engagement on health outcomes is substantial, with evidence that engaged patients are more likely to adhere to treatment regimens, show greater self-efficacy, and adopt beneficial lifestyle modifications that collectively improve long-term cardiovascular health.^59^ A central debate is whether reducing future cardiovascular disease risk can be achieved solely through effective postpartum blood pressure control, or whether antenatal management might also mitigate long-term cardiovascular consequences. Our systematic review shows a total absence of studies assessing antenatal interventions with extended follow-up, restricting conclusions.

Pharmaceutical and nutraceutical interventions delivered exclusively postpartum showed no significant reduction in systolic and diastolic blood pressure. There is little evidence on whether these changes were sustained. A significant reduction in blood pressure might result from continuous antihypertensive therapy spanning the antenatal and postnatal periods;^60^ however, no studies were identified, despite using search terms designed to capture such studies. The first week postpartum has been identified as a critical period of cardiovascular remodelling that, if appropriately managed, appears to relate to long-term outcomes.^43^ However, most studies did not report postpartum follow-up data, limiting the evaluation of long-term cardiovascular risk. Choice of medication and how aggressively the hypertension is managed could both determine future risk of cardiovascular disease, similar to findings from studies in older, non-pregnant populations.^61^ No studies assessed optimal blood pressure control; work is needed in this area. Therefore, whether effective antenatal blood pressure management alone can sufficiently reduce future cardiovascular risk is unclear. The total absence of validated surrogate markers in interventional studies also limited our ability to determine whether interventions might affect cardiovascular phenotypes relevant to long-term cardiovascular risk.

Because most studies reported clinical outcomes up to 2 weeks postpartum, this might lead to an underestimation of the potential long-term effects of interventions performed peripartum. The longest follow-up identified was from a postpartum clinic monitoring women with preterm pre-eclampsia over 10 years.^49^ Women who attended a clinic and received individualised postpartum lifestyle advice had a lower incidence of hypertension. However, the absence of more detailed risk stratification, such as lifestyle data or biomarker analysis, limits the interpretation of the results in terms of behavioural influence and underlying risk modification. Across all outcomes, few studies were powered to detect differences in rare events, and most findings were based on small absolute numbers.

Our systematic review highlights a disconnect between the management of hypertensive disorders during pregnancy and the subsequent strategies aimed at long-term cardiovascular risk reduction and prevention. The predominant models linking hypertensive disorders of pregnancy with later-life cardiovascular disease consistently implicate underlying maternal cardiovascular and vascular abnormalities that might be modifiable and continue to be evident after pregnancy.^62^, ^63^ These abnormalities could include both subclinical vascular or cardiac injury during pregnancy and pre-existing vascular or cardiac dysfunction that predisposes women to both disorders of pregnancy and cardiovascular disease. Only 17 (53%) of the 32 included studies reported an observation period extending beyond 1 week postpartum, and most relied solely on blood pressure as a surrogate marker of cardiovascular risk. Design of future clinical trials investigating hypertensive disorders of pregnancy should routinely incorporate strategies to evaluate how therapies delivered during pregnancy affect future cardiovascular status (appendix 1 [p 43]). A core outcome set is invaluable and would improve comparability across studies as well as recognise clinical relevance and strengthen patient-centred care.

There was scarce evidence of explicit consideration of race, ethnicity, geography, and socioeconomic status in trial design. Trials could be designed with recognition that social determinants of health substantially contribute to risk of cardiovascular disease, with pre-eclampsia linked to lower socioeconomic status.^64^ With rising evidence of haemodynamic differences in the spectrum of hypertensive disorders in pregnancy, definitions should be consistent in trials, particularly as these guide treatment selection and would allow a meaningful network meta-analysis.^65^, ^66^

Of the 32 studies included in the systematic review, 11 (34%) had interventions performed in the second and third trimester. The other studies with postnatal follow-up had interventions commenced in the immediate postpartum period. Of the 90 studies excluded due to no postpartum follow-up, 76 (84%) performed interventions in the third trimester, with 14 (16%) studies including women in the second trimester. The timing of intervention is probably due to the distinction between the acute treatment of blood pressure and its immediate effects on perinatal morbidity and reduction of ongoing cardiovascular risk. Clinical trials that investigate how interventions initiated at each trimester affect long-term cardiovascular outcomes would contribute to a more robust evidence base.

The interventions assessed in this systematic review encompassed lifestyle, pharmaceutical and nutraceutical therapy, and remote blood pressure management approaches. 25 (78%) studies evaluated pharmaceutical and nutraceutical interventions, while only four (13%) addressed lifestyle modifications, and three (9%) investigated remote blood pressure management. Emerging technologies that facilitate the delivery of lifestyle modifications with blood pressure management during the postpartum period could offer accessible solutions. Among the pharmaceutical and nutraceutical interventions, only enalapril was evaluated for postpartum benefit.^46^ The observed improvement in cardiac function might reflect enalapril's ability to halt the progression of hypertension—a benefit that has been shown in non-pregnant populations.^67^

Although blood pressure is an accessible measure of cardiovascular stress, assessment of other risk markers, including lipid profiles, smoking status, diabetes, and inflammatory status, might provide a more holistic evaluation.^68^ Consent of participants to make use of routinely collected health record data, or for participation in a observational cohort study, could also allow for long-term monitoring of the effects of interventions during the peripartum period. The use of angiogenic markers might give insight into the pathophysiology and long-term sequelae of pre-eclampsia, particularly with regard to endothelial dysfunction.^69^ Placental growth factor (a pivotal angiogenic mediator) has emerged as a potential marker of future cardiovascular risk, with reduced concentrations linked to persistent adverse cardiovascular phenotypes.^70^ However, its prognostic utility remains uncertain, and research is needed to establish whether placental growth factor deficiency serves as a reliable determinant of long-term cardiovascular disease.^71^ Importantly, interventional studies suggest complexity in this relationship: a prolonged course of enalapril did not influence circulating placental growth factor concentrations, yet improved left-ventricular remodelling.^46^

However, traditional cardiac biomarkers appear to have limited use in this population. Postpartum studies have reported no significant differences in circulating cardiac troponin or NT-proBNP between women with a history of pre-eclampsia and controls, despite echocardiographic evidence of sustained structural changes, such as increased left ventricular mass.^46^, ^72^ This dissociation suggests that hypertrophic remodelling in this context does not follow the conventional patterns of myocyte injury. Therefore, alternative biomarkers might be more informative. For example, elevated concentrations of GDF-15 have been observed 1–3 years postpartum in women with early onset pre-eclampsia, implicating its potential role in maladaptive cardiac remodelling.^72^

In addition to biochemical indicators, physiological markers, such as arterial stiffness, might be of value in populations lacking traditional cardiovascular risk factors.^73^ Imaging-based outcomes have been validated as predictive markers for 10-year cardiovascular risk, reinforcing their use in long-term monitoring following hypertensive disorders of pregnancy.^74^ Women with previous hypertensive disorders of pregnancy show persistent structural heart changes, including left-ventricular remodelling and carotid thickening.^11^, ^75^ Studies have reported concentric remodelling, eccentric hypertrophy, and left-atrial enlargement up to a decade following postpartum,^76^, ^77^ as well as feasibility of interventions to affect concentric remodelling, left ventricular hypertrophy, and diastolic dysfunction.^43^, ^46^

The systematic review has some limitations. 85 studies were excluded due to no postpartum follow-up. The majority of these studies were pharmaceutical trials that restricted their outcomes to antenatal blood pressure measurements. Our inclusion criteria also excluded studies performing interventions in women at risk of pre-eclampsia. These studies might have addressed underlying cardiac or vascular dysfunction and included postpartum follow-up.

The breadth of interventions evaluated across the included studies resulted in substantial heterogeneity that limited the feasibility of statistical pooling. Similarly, the diversity in cardiovascular outcomes assessed restricted the scope for meaningful interpretation and direct comparison of results. Only RCTs were included in the meta-analyses because these studies satisfied the inclusion criteria. The initial search strategy was intentionally broad and not restricted to RCTs to capture the full extent of available evidence. Although the case-control study identified was not eligible for inclusion in the meta-analyses, it was retained in the systematic review because of its extended duration of follow-up.^49^ We acknowledge the limitation of including a case-control study in which unmeasured confounding and selection bias remain possible. Women who attend specialist postpartum hypertension clinics might be more motivated to engage in health-promoting behaviours, such as weight management, which could independently reduce cardiovascular risk. Finally, the geographical representation of studies was restricted, with only one study done in a low-income country (Sudan). The poor representation of low-income country diversity is a crucial gap because hypertensive disorders of pregnancy carry particularly high morbidity and mortality in these settings.

Furthermore, studies using active control groups or controls consisting of women without hypertensive disorders of pregnancy were excluded to maintain comparability within the study populations. Our systematic review was also restricted to trials done specifically in women diagnosed with hypertensive disorders of pregnancy. As a result, broader cardiovascular trials involving general female populations, which might have included subsets of women with a history of hypertensive pregnancy disorders, were not captured, potentially limiting the generalisability of our findings to other relevant cohorts.

There were no high-quality RCTs that assessed the long-term cardiovascular effects of interventions around the time of pregnancy in women with hypertensive disorders of pregnancy. No studies reported interventions that bridged the antenatal and postnatal periods, which might reflect fragmented continuity of care and transitions between obstetric, primary care, and cardiology services. Nonetheless, the available trials do show some benefit. Our meta-analysis suggests that remote blood pressure management appears to be the most effective intervention for reducing both systolic and diastolic blood pressure, with less variability compared with pharmaceutical and nutraceutical interventions. Given the well established link between hypertensive disorders of pregnancy and future cardiovascular morbidity, research that focuses on long-term cardiovascular follow-up, which might include validated surrogate markers of cardiovascular disease, should be prioritised to reduce the global burden of cardiovascular disease in women. We have identified key knowledge gaps that could be explored to gain insight into which interventions most effectively reduce lifetime cardiovascular risk. The increasing recognition of adverse pregnancy outcomes as sex-specific cardiovascular risk factors highlights the peripartum period as a potentially crucial window for preventive strategies.

### Contributors

### Data sharing

No new data were generated for this meta-analysis. All data analysed were extracted from published studies and are available from the original publications cited in this Article. Requests for access to individual participant data should be directed to the corresponding authors of the original studies, in accordance with their data sharing policies.

## Declaration of interests

JK has received support for attending meetings from the British Society of Cardiovascular Imaging/British Society of Cardiac Computed Tomography (BSCI/BSCCT) and is the accreditation lead for BSCI/BSCCT. PL has received grants or contracts from the UK Medical Research Council, UK National Institute of Health and Social Care Research, British Heart Foundation, Merck & Cie, Osler Diagnostics, Ultromics, and Novo Nordisk; is a founder and shareholder of Ultromics; has received consulting fees from Ultromics, speaker fees from Pfizer and Bracco, and advisory board fees from Daiichi Sankyo and AstraZeneca; is a named inventor on three granted patents and one filed patent related to artificial intelligence and imaging; and is the chair of the UK National Institute for Health and Care Research (NIHR) i4i Product Development Awards Funding Panel and a past member of the UK National Institute for Health and Care Excellence Cardiovascular Prevention Guideline Committee. All other authors declare no competing interests.
