# Supplementary material for: Peripartum interventions for elevated blood pressure in women with hypertensive disorders of pregnancy: a systematic review and meta-analysis
Source: Lancet Obstet Gynaecol Womens Health. 2026 May;2(5):e435–50. doi: 10.1016/S3050-5038(25)00183-9 (PMC13151740; doi:10.1016/S3050-5038(25)00183-9)
Supplement: Supplementary appendix 1 [file mmc1.pdf]

THE LANCET
Obstetrics, Gynaecology, & Women's Health

Supplementary appendix 1

This appendix formed part of the original submission and has been peer reviewed. We post it as supplied by the authors.

Supplement to: Frost AL, Krasner S, Li M, et al. Peripartum interventions for elevated blood pressure in women with hypertensive disorders of pregnancy: a systematic review and meta-analysis. *Lancet Obstet Gynaecol Womens Health* 2026; published online March 19. https://doi.org/10.1016/S3050-5038(25)00183-9.

Peripartum interventions for elevated blood pressure in women with hypertensive disorders of pregnancy: a systematic review and meta-analysis of postpartum cardiovascular outcomes

Supplementary File Contents

**Item**	**Page(s)**
**MATERIALS**	
Supplementary Materials 1A – PRIMSA checklist	2–4
Supplementary Materials 1B – PRISMA abstract checklist	5
Supplementary Materials 2 – Search Strategies	6–8
Supplementary Materials 3 – Risk of bias assessments	9–10
**TABLES**	
Supplementary Table 1 – Terminology and examples of categorisation of intervention	11
Supplementary Table 2 – List of studies excluded during full-text screening	12–31
Supplementary Table 3 – GRADE assessment	32–33
Supplementary Table 4 – Further details describing study populations and interventions	34–42
Supplementary Table 5 – Recommendations for future research	43
**FIGURES**	
Supplementary Figure 1 – Follow up time points for reported outcomes	44
Supplementary Figure 2 – Leave-one-out sensitivity analysis	45–46
Supplementary Figure 3 – Funnel plots for publication bias assessment	47

SUPPLEMENTARY MATERIALS 1A

Section and Topic	Item #	Checklist item	Location where item is reported
**TITLE**			
Title	1	Identify the report as a systematic review.	Page (P) 1
**ABSTRACT**			
Abstract	2	See the PRISMA 2020 for Abstracts checklist.	Supplementary Materials 1b
**INTRODUCTION**			
Rationale	3	Describe the rationale for the review in the context of existing knowledge.	P3, 6
Objectives	4	Provide an explicit statement of the objective(s) or question(s) the review addresses.	P6
**METHODS**			
Eligibility criteria	5	Specify the inclusion and exclusion criteria for the review and how studies were grouped for the syntheses.	P7-8, Supplementary Materials 2
Information sources	6	Specify all databases, registers, websites, organisations, reference lists and other sources searched or consulted to identify studies. Specify the date when each source was last searched or consulted.	P7
Search strategy	7	Present the full search strategies for all databases, registers and websites, including any filters and limits used.	Supplementary Materials 2
Selection process	8	Specify the methods used to decide whether a study met the inclusion criteria of the review, including how many reviewers screened each record and each report retrieved, whether they worked independently, and if applicable, details of automation tools used in the process.	P7-8
Data collection process	9	Specify the methods used to collect data from reports, including how many reviewers collected data from each report, whether they worked independently, any processes for obtaining or confirming data from study investigators, and if applicable, details of automation tools used in the process.	P8, Supplementary Materials 2
Data items	10a	List and define all outcomes for which data were sought. Specify whether all results that were compatible with each outcome domain in each study were sought (e.g. for all measures, time points, analyses), and if not, the methods used to decide which results to collect.	P7
	10b	List and define all other variables for which data were sought (e.g. participant and intervention characteristics, funding sources). Describe any assumptions made about any missing or unclear information.	Supplementary Materials 2
Study risk of bias assessment	11	Specify the methods used to assess risk of bias in the included studies, including details of the tool(s) used, how many reviewers assessed each study and whether they worked independently, and if applicable, details of automation tools used in the process.	P8, Supplementary Materials 3
Effect measures	12	Specify for each outcome the effect measure(s) (e.g. risk ratio, mean difference) used in the synthesis or presentation of results.	P8

Section and Topic	Item #	Checklist item	Location where item is reported
Synthesis methods	13a	Describe the processes used to decide which studies were eligible for each synthesis (e.g. tabulating the study intervention characteristics and comparing against the planned groups for each synthesis (item #5)).	P8
	13b	Describe any methods required to prepare the data for presentation or synthesis, such as handling of missing summary statistics, or data conversions.	P8
	13c	Describe any methods used to tabulate or visually display results of individual studies and syntheses.	P8
	13d	Describe any methods used to synthesize results and provide a rationale for the choice(s). If meta-analysis was performed, describe the model(s), method(s) to identify the presence and extent of statistical heterogeneity, and software package(s) used.	P8
	13e	Describe any methods used to explore possible causes of heterogeneity among study results (e.g. subgroup analysis, meta-regression).	P8
	13f	Describe any sensitivity analyses conducted to assess robustness of the synthesized results.	P8
Reporting bias assessment	14	Describe any methods used to assess risk of bias due to missing results in a synthesis (arising from reporting biases).	P8, Supplementary Materials 3
Certainty assessment	15	Describe any methods used to assess certainty (or confidence) in the body of evidence for an outcome.	P8, Supplementary Table 3
**RESULTS**			
Study selection	16a	Describe the results of the search and selection process, from the number of records identified in the search to the number of studies included in the review, ideally using a flow diagram.	P9, Figure 1
	16b	Cite studies that might appear to meet the inclusion criteria, but which were excluded, and explain why they were excluded.	Supplementary Table 2
Study characteristics	17	Cite each included study and present its characteristics.	Table 1, Supplementary Table 4
Risk of bias in studies	18	Present assessments of risk of bias for each included study.	Supplementary Materials 3
Results of individual studies	19	For all outcomes, present, for each study: (a) summary statistics for each group (where appropriate) and (b) an effect estimate and its precision (e.g. confidence/credible interval), ideally using structured tables or plots.	P9-11, Figures 2-3, Supplementary Table 3
Results of syntheses	20a	For each synthesis, briefly summarise the characteristics and risk of bias among contributing studies.	P9, Supplementary Materials 3
	20b	Present results of all statistical syntheses conducted. If meta-analysis was done, present for each the summary estimate and its precision	P9-10, Figures

Section and Topic	Item #	Checklist item	Location where item is reported
		(e.g. confidence/credible interval) and measures of statistical heterogeneity. If comparing groups, describe the direction of the effect.	2-3
	20c	Present results of all investigations of possible causes of heterogeneity among study results.	P10, Supplementary Figures 2-3
	20d	Present results of all sensitivity analyses conducted to assess the robustness of the synthesized results.	P10, Supplementary Figures 2-3
Reporting biases	21	Present assessments of risk of bias due to missing results (arising from reporting biases) for each synthesis assessed.	Supplementary Materials 3
Certainty of evidence	22	Present assessments of certainty (or confidence) in the body of evidence for each outcome assessed.	P9, Supplementary Table 3
**DISCUSSION**			
Discussion	23a	Provide a general interpretation of the results in the context of other evidence.	P3, 13-16
	23b	Discuss any limitations of the evidence included in the review.	P16-17
	23c	Discuss any limitations of the review processes used.	P16-17
	23d	Discuss implications of the results for practice, policy, and future research.	P3, 14-15, 17-18, Supplementary Table 5
**OTHER INFORMATION**			
Registration and protocol	24a	Provide registration information for the review, including register name and registration number, or state that the review was not registered.	P5, 8
	24b	Indicate where the review protocol can be accessed, or state that a protocol was not prepared.	P5, 8
	24c	Describe and explain any amendments to information provided at registration or in the protocol.	N/A
Support	25	Describe sources of financial or non-financial support for the review, and the role of the funders or sponsors in the review.	P5
Competing interests	26	Declare any competing interests of review authors.	P2
Availability of data, code and other materials	27	Report which of the following are publicly available and where they can be found: template data collection forms; data extracted from included studies; data used for all analyses; analytic code; any other materials used in the review.	P2

From: Page MJ, McKenzie JE, Bossuyt PM, Boutron I, Hoffmann TC, Mulrow CD, et al. The PRISMA 2020 statement: an updated guideline for reporting systematic reviews. BMJ 2021;372:n71. doi: 10.1136/bmj.n71. This work is licensed under CC BY 4.0. To view a copy of this license, visit https://creativecommons.org/licenses/by/4.0/

SUPPLEMENTARY MATERIALS 1B

Section and Topic	Item #	Checklist item	Reported (Yes/No)
**TITLE**			
Title	1	Identify the report as a systematic review.	Yes
**BACKGROUND**			
Objectives	2	Provide an explicit statement of the main objective(s) or question(s) the review addresses.	Yes
**METHODS**			
Eligibility criteria	3	Specify the inclusion and exclusion criteria for the review.	Yes
Information sources	4	Specify the information sources (e.g. databases, registers) used to identify studies and the date when each was last searched.	Yes
Risk of bias	5	Specify the methods used to assess risk of bias in the included studies.	Yes
Synthesis of results	6	Specify the methods used to present and synthesise results.	Yes
**RESULTS**			
Included studies	7	Give the total number of included studies and participants and summarise relevant characteristics of studies.	Yes
Synthesis of results	8	Present results for main outcomes, preferably indicating the number of included studies and participants for each. If meta-analysis was done, report the summary estimate and confidence/credible interval. If comparing groups, indicate the direction of the effect (i.e. which group is favoured).	Yes
**DISCUSSION**			
Limitations of evidence	9	Provide a brief summary of the limitations of the evidence included in the review (e.g. study risk of bias, inconsistency and imprecision).	Yes
Interpretation	10	Provide a general interpretation of the results and important implications.	Yes
**OTHER**			
Funding	11	Specify the primary source of funding for the review.	Yes
Registration	12	Provide the register name and registration number.	Yes

From: Page MJ, McKenzie JE, Bossuyt PM, Boutron I, Hoffmann TC, Mulrow CD, et al. The PRISMA 2020 statement: an updated guideline for reporting systematic reviews. BMJ 2021;372:n71. doi: 10.1136/bmj.n71. This work is licensed under CC BY 4.0. To view a copy of this license, visit https://creativecommons.org/licenses/by/4.0/

SUPPLEMENTARY MATERIALS 2

Search Terms

Full search terms used to query Ovid are detailed in the table below. Four separate sub-queries were designed to include search terms relevant to population (#1), intervention (#2, outcomes (#3), and study design (#4). These sub-queries were combined to form the overall search terms used for the systematic review (#5).

Number	Search Terms
#1	exp "hypertension, pregnancy-induced"/ or exp pre-eclampsia/ or ("hypertensive disorder* of pregnancy" or "hypertensive pregnanc*" or preeclamp* or pre-eclamp* or preclamp* or "edema proteinuria hypertension gestosis" or "proteinuria edema hypertension gestosis" or "eph complex" or "eph toxemia" or "eph toxaemia" or "eph gestosis" or "toxemia of pregnanc*" or "toxaemia of pregnanc*" or eclamp* or HELLP or "h?emolysis, elevated liver enzymes*" or ((pregnan* or gestation*) adj3 (h?emolysis or "elevated liver enzymes")) or "gestational hypertensi*" or "pregnancy-induced hypertension").ti,ab.
#2	exp "cardiovascular agents"/ or aspirin/ or exp biopterin/ or exp "folic acid"/ or exp tetrahydrofolates/ or exp vitamins/ or exp provitamins/ or exp vitamin b complex/ or "sildenafil citrate".mp. or exp pharmaceutical preparations/ or exp dosage forms/ or capsules/ or exp colloids/ or exp drug combinations/ or nonprescription drugs/ or pharmaceutic aids/ or exp illicit drugs/ or pharmacologic actions/ or "specialty uses of chemicals"/ or Calcium/ or exp Hydroxymethylglutaryl-CoA Reductase Inhibitors/ or exp Metformin/ or exp Steroids/ or betamethasone.mp. or magnesium.ti,ab. or "Self monitoring".mp. or "telemonitor*".mp. or " self titration".mp. or "self management".mp. or (self adj3 (monitor* or titration or medica*)).mp. or telemetr*.mp. or "remote sensing technolog*".mp. or ((patient or continuous or wireless or surveillance) adj3 monitoring).mp. or exp telemetry/ or exp remote sensing technology/ or exp Life Style/ or exp "diet, food, and nutrition"/ or diet.ti,ab. or motor activity/ or exercise/ or physical conditioning, human/ or running/ or swimming/ or walking/
#3	exp "magnetic resonance imaging"/ or exp ultrasonography/ or exp "pulse wave analysis"/ or "magnetic resonance".mp. or "MR imaging".mp. or MRI.mp. or "cardiac MR*".mp. or "cardiovascular MR*".mp. or "diffusion tensor imaging".mp. or "diffusion weight* imaging".mp. or "diffusion imaging".mp. or echocardiograph*.mp. or cardioechograph*.mp. or echocardiogram.mp. or "cardiac structure*".mp. or "myocardial structure*".mp. or "cardiac function*".mp. or "myocardial function*".mp. or microvascula* macrovascula*.mp. or macrovessel* exp heart disease risk factors/ or "blood pressure".mp. or hypertension.mp. or hypercholesterolaemia.mp. or (cholesterol adj3 (low or high)).mp. or "high density lipoprotein".mp. or "low density lipoprotein".mp. or dyslipidemia.mp. or Obesity/ or Obesity, Maternal/ or

	Obesity, Abdominal/ or Diabetes Mellitus, Type 1/ or Diabetes Mellitus, Type 2/ or "QRisk".mp. or "QRISK2.mp. or "SCORE2".mp. or Framingham.mp. or exp plaque, atherosclerotic/ or exp vascular remodeling/ or exp ventricular remodeling/ or exp heart diseases/ or exp vascular diseases/ or "heart failure".mp. or "cardiovascular disease".mp. or "heart disease".mp. or "myocardial infarction".mp. or angina.mp. or "cerebrovascular event".mp. or stroke.mp. or "renal failure".mp. or "kidney failure".ti,ab.
#4	("randomized controlled trial" OR "controlled clinical trial" OR "pragmatic clinical trial" OR "clinical study" OR "adaptive clinical trial" OR "equivalence trial" OR "clinical trial" OR "multicenter study").pt. OR exp "randomized controlled trials as topic"/ OR exp "controlled clinical trials as topic"/ OR "clinical studies as topic"/ OR exp "clinical trial"/ OR exp "clinical trials as topic"/ OR exp "multicenter study"/ OR "multicenter studies as topic"/ OR exp "random allocation"/ OR randomization/ OR exp "double-blind method"/ OR "double blind procedure"/ OR "double-blind studies"/ OR exp "single-blind method"/ OR "single blind procedure"/ OR "single-blind studies"/ OR placebos/ OR placebo/ OR exp "control groups"/ OR "cross-over studies"/ OR "crossover procedure"/ OR (random* OR sham OR placebo*).tw,kf. OR ((singl* OR doubl*) ADJ (blind* OR dumm* OR mask*)).tw,kf. OR ((tripl* OR trebl*) ADJ (blind* OR dumm* OR mask*)).tw,kf. OR (control* ADJ3 (study OR studies OR trial* OR group*)).tw,kf. OR (clinical ADJ3 (study OR studies OR trial*)).tw,kf. OR (nonrandom* OR non-random* OR quasi-random* OR quasirandom*).tw,kf. OR (phase ADJ3 (study OR studies OR trial*)).tw,kf. OR ((crossover OR cross-over) ADJ3 (study OR studies OR trial*)).tw,kf. OR ((multicent* OR multi-cent*) ADJ3 (study OR studies OR trial*)).tw,kf. OR allocated.tw,kf. OR ("open label" ADJ5 (study OR studies OR trial*)).tw,kf. OR ((equivalence OR superiority OR non-inferiority OR noninferiority) ADJ3 (study OR studies OR trial*)).tw,kf. OR ("pragmatic study" OR "pragmatic studies").tw,kf. OR ((pragmatic OR practical) ADJ3 trial*).tw,kf. OR "quasi experimental methods"/ OR "quasi experimental study"/ OR ((quasiexperimental OR quasi-experimental) ADJ3 (study OR studies OR trial*)).tw,kf.
#5	#1 and #2 and #3 and #4

Study Selection

Inclusion Criteria

The inclusion criteria were as follows: (1) Original research articles in humans; (2) Studies reporting cardiovascular outcomes in mothers with hypertensive disorders of pregnancy without time limitation for follow-up period; (3) Cardiovascular outcomes reported include (a) cardiovascular risk factors including blood pressure, levels of HDL and non-HDL cholesterol (dyslipidaemia), BMI, new diagnoses of diabetes (b) measures of cardiac structure and vascular function including right and left ventricular mass, right and left

ventricular systolic and diastolic function, angiography, carotid imaging, atheroma formation (c) development of cardiac and vascular disease such as heart failure, myocardial infarction, angina, stroke and renal failure; (3) Studies perform comparison against those without intervention.

Data Extraction

Using standardised forms, the following data were extracted from included studies: first author, year of publication, country, study design, setting, years recorded, total number of patients with HDP, type of HDP, definition used to determine HDP, inclusion and exclusion criteria, number of women in the intervention and control arm, duration of observation period, population demographics, intervention type and details of administration, outcome measures, timing of measurement post intervention, timing of follow up, secondary outcome measures, adverse events and randomisation methods.

SUPPLEMENTARY MATERIALS 3

Risk of Bias Assessment

Methodology – Risk of bias assessment for 31 RCTs was conducted independently by two reviewers (AF and SK) using the Cochrane Risk of Bias Tool (RoB 2). This tool evaluates bias across five key domains: (1) bias arising from the randomisation process, (2) deviations from intended interventions, (3) missing outcome data, (4) measurement of the outcome, and (5) selection of the reported result. Each study was rated as low risk, some concerns, or high risk of bias based on predefined criteria. Discrepancies between reviewers were resolved through discussion or consultation with a third reviewer (ML) when necessary. The overall risk of bias assessment for each study was summarised in a risk of bias table and visualised using a summary plot.

Results – Most studies (61%) were categorised as having "some concerns," primarily due to the lack of a published protocol or statistical analysis plan (Figure A). The risk of bias in the randomisation process was generally low, with 28 out of 31 studies demonstrating adequate random sequence generation and allocation concealment. However, one study was identified as having a high risk of bias, as all study personnel were unblinded to group assignments.

Overall, three studies were judged to have a high risk of bias due to multiple factors, including unblinded allocation of groups, deviations from the intended intervention, missing outcome data, and insufficient detail in the measurement of outcomes. In contrast, nine out of thirty-one studies were classified as having a low risk of bias across all domains, indicating robust methodology.

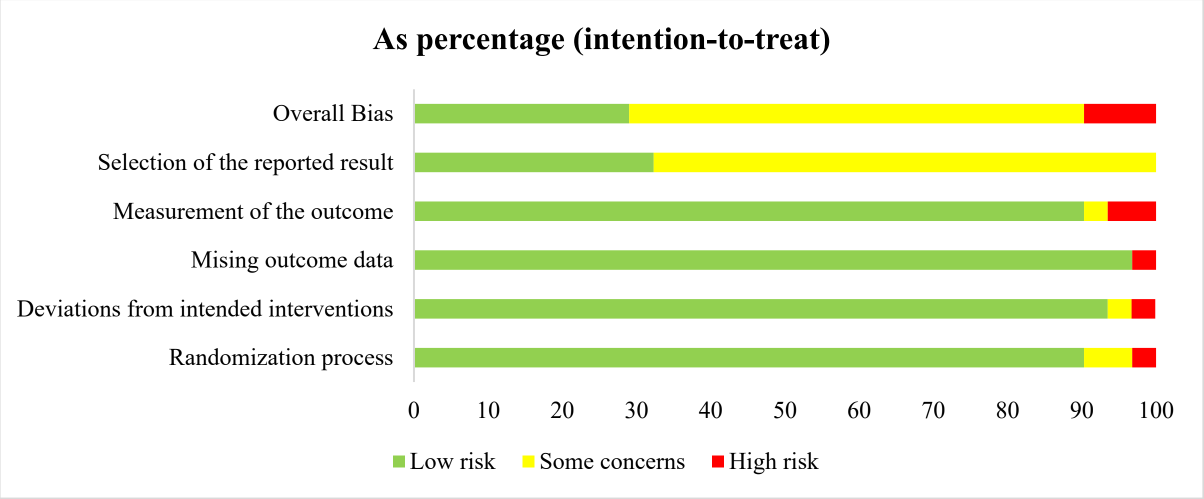

Figure A. Proportion of studies at each level of bias across key domains as assessed using the Cochrane Risk of Bias 2 (RoB 2) tool, presented as percentages under the intention-to-treat analysis. The chart displays six domains of bias: the randomisation process, deviations from intended interventions, missing outcome data, measurement of the outcome, selection of the reported result, and overall bias. Each domain is color-coded to indicate the risk level: green for low risk of bias, yellow for some concerns, and red for high risk of bias.

SUPPLEMENTARY TABLE 1

Terminology and examples of categorisation of intervention.

PROSPERO Categorisation	Manuscript Categorisation	Examples of Interventions
'Exercise such as physical conditioning, running, walking, swimming and adjustments to food, diet and nutrition'	Lifestyle	Recommendations or action plans for modifiable risk factors such as dietary advice, physical activity plans/ motivation, stress management, weight management. We also included the use of a neonatal sleep aid under lifestyle due to the effect of increased infant sleep on maternal sleep deprivation.
'Drug therapy- Cardiovascular and antihypertensive agents inclusive but not limited to beta blockers, calcium channel blockers, metformin, steroids. Non prescriptive preparations and vitamins.'	Pharmaceutical and Nutraceutical Therapy	Antihypertensive therapy, steroids, analgesia, as well as prescribed nutritional supplements, such as amino acids (e.g. l-arginine) and probiotics.
'Telemetry- self management of blood pressure and/or physician surveillance'	Remote Blood Pressure Management	Participants are provided with a validated blood pressure monitor with secure telemonitoring. All trials incorporated management when readings were above the normal/expected threshold.

SUPPLEMENTARY TABLE 2

List of all papers excluded during full-text screening with reason for exclusion given for each.

	**Excluded Titles**	**Author**	**Reasons for exclusion**
1	A comparative study to evaluate the role of alpha tocopherol in pregnancy induced hypertension	Bashir	Full text requested from authors
2	A comparison of nifedipine with methyldopa for control of blood pressure in mild to moderate pregnancy induced hypertension	Bharathi	Full text requested from authors
3	Nifedipine influence on lipid and some antioxidant parameters in pregnancy induced hypertension	Ungureanu	Full text requested from authors
4	A comparative randomised controlled parallel group study of maternal, fetal and neonatal outcomes of labetalol versus methyldopa in the treatment of new onset hypertension during pregnancy	Verma	Full text requested from authors
5	Impact of Alkaline Ionized Water and Nutritional Supplement on Progression of Pregnancy Induced Hypertension: Randomized Control Trial	Adel O	Unable to access full text
6	Intravenous labetalol versus oral nifedipine in the treatment of severe hypertension in pregnancy	Devi Mayanglambam R	Unable to access full text
7	Utilization of hydralazine or alpha-methyldopa for the management of early puerperal hypertension	Griffis	Unable to access full text

8	The role of intravenous volume expansion in moderate pre-eclampsia	Heilmann	Unable to access full text
9	A Prospective Controlled Trial Of Metoprolol-Hydralazine Treatment In Hypertension During Pregnancy	Hogstedt	Unable to access full text
10	A comparison of nifedipine with methyldopa in pregnancy induced hypertension	Jayawardana	Unable to access full text
11	Effect of L-arginine supplementation in pregnant women with chronic hypertension and previous placenta vascular disorders receiving Aspirin prophylaxis: a randomized control trial	Monari	Unable to access full text
12	Efficacy of Antithrombin Replacement Therapy in Severe Early-Onset Preeclampsia	Nakabayashi	Unable to access full text
13	A comparison of antihypertensive efficacy of methyldopa versus labetalol in the treatment of pregnancy-induced hypertension	Qazi	Unable to access full text
14	Ketanserin and hydralazine in hypertension in pregnancy--a randomised double-blind trial	Rossouw	Unable to access full text
15	The effect of magnesium on maternal blood pressure in pregnancy-induced hypertension A randomized double-blind placebo-controlled trial	Rudnicki	Unable to access full text
16	Progesterone Therapy in Pre-Eclamptic Toxaemia	Sammour	Unable to access full text
17	Clinical Effect of Nimodipine Combined with Magnesium Sulfate on Pregnancy-Induced Hypertension Syndrome	Chang	Retracted
18	Effect of Dexmedetomidine on Cardiac Output among Parturient with Severe Preeclampsia after Cesarean Section	Lv	Retracted

19	Nifedipine plus Systematic Nursing on Pregnancy Outcomes in Patients with Gestational Hypertension	Ma	Retracted
20	Effects of Low-Dose Aspirin Combined with Vitamin E on the Incidence of Intrauterine Growth Restriction and Hemorheological Indexes of Pregnant Women in Patients with Gestational Hypertension	Shan	Retracted
21	Effects of Nifedipine and Labetalol Combined with Magnesium Sulfate on Blood Pressure Control, Blood Coagulation Function, and Maternal and Infant Outcome in Patients with Pregnancy-Induced Hypertension	Shao	Retracted
22	The effect of magnesium sulfate treatment on serum cardiac troponin I levels in preeclamptic women	Atalay	No comparator/control group
23	Effect of stretching exercises versus autogenic training on preeclampsia	Awad	No comparator/control group
24	Blood pressure monitoring and knowledge in the first year after a hypertensive disorder of pregnancy	Cameron	No comparator/control group
25	S-Nitrosoglutathione improves haemodynamics in early-onset pre-eclampsia	Everett	No comparator/control group
26	A new treatment of severe pre-eclampsia by long-term epidural anaesthesia	Kanayama	No comparator/control group
27	Labetalol, Low-dose Aspirin, and Vitamin E and Calcium for Gestational Hypertension and Influence on MicroRNA-126 and PLGF Levels	Kou	No comparator/control group

28	Clinical Outcomes Associated With a Remote Postpartum Hypertension Monitoring Program	Lemon	No comparator/control group
29	The effect of loading dose of magnesium sulfate on uterine, umbilical, and fetal middle cerebral arteries Doppler in women with severe preeclampsia: A case control study	Maged	No comparator/control group
30	Methyldopa versus nifedipine or no medication for treatment of chronic hypertension during pregnancy: A multicenter randomized clinical trial	Salama	No comparator/control group
31	Effect of short-term changes in salt intake on plasma cytokines in women with healthy and hypertensive pregnancies	Thangaraj	No comparator/control group
32	Control of preeclamptic hypertension by ketanserin, a new serotonin receptor antagonist	Weiner	No comparator/control group
33	Differential effects of renin-angiotensine-aldosteron system inhibition, sympathoinhibition and low sodium diet on blood pressure in women with a history of preeclampsia: A double-blind, placebo-controlled cross-over trial (the PALM study)	Zoet	No comparator/control group
34	Corticosteroid therapy for prevention of respiratory distress syndrome in severe preeclampsia	Amorim	Wrong outcome
35	The Magpie Trial: a randomised trial comparing magnesium sulphate with placebo for pre-eclampsia. Outcome for children at 18 months	Duley	Wrong outcome
36	Oral antihypertensive regimens (nifedipine retard, labetalol,and methyldopa) for management of severe hypertension in pregnancy: an open-label, randomised controlled trial	Easterling	Wrong outcome

37	Dexamethasone for the treatment of class I HELLP syndrome: A double- blind, placebo-controlled, multicenter, randomized clinical trial	Fonseca	Wrong outcome
38	Effect of the Beta-Adrenergic Blocker Pindolol on Platelet Function in Chronic Hypertensive Pregnancy	Hirsch	Wrong outcome
39	Uterine Ultrasound Doppler Hemodynamics of Magnesium Sulfate Combined with Labetalol in the Treatment of Pregnancy-Induced Hypertension Using Empirical Wavelet Transform Algorithm	Liu	Wrong outcome
40	Control of Hypertension In Pregnancy Study randomised controlled trial—are the results dependent on the choice of labetalol or methyldopa?	Magee	Wrong outcome
41	A randomized controlled trial of complete bed rest versus ambulation in the management of proteinuric hypertension during pregnancy	Mathews	Wrong outcome
42	Effects of acute L-arginine infusion on non-stress test in hypertensive pregnant women	Neri	Wrong outcome
43	Furosemide for postpartum blood pressure control in patients with hypertensive disorders	Pagan	Wrong outcome
44	Uterine and fetal hemodynamics and fetal cardiac function after atenolol and pindolol infusion. A randomized study	Rasanen	Wrong outcome
45	Effect of methyldopa and isradipine on fetal heart rate pattern assessed by computerized cardiotocography in human pregnancy	Swensson	Wrong outcome

46	Efficacy of nifedipine tablets plus aspirin in patients with gestational hypertension and the effect on coagulation function and hemorheology	Yin	Wrong outcome
47	Management Of Hypertension In The Early Postpartum: A Randomized Controlled Trial	Bortolotto	Conference abstract
48	A comparison of phentolamine and magnesium sulfate therapy in pre-eclampsia	Dianrong	Conference abstract
49	Clonidine versus captopril for severe postpartum hyper- tension: A randomized controlled trial	Katz	Conference abstract
50	The control of hypertension in pregnancy study (CHIPS) randomized controlled trial.	Magee	Conference abstract
51	Does antihypertensive therapy during pregnancy increase postpartum medication adherence for women with mild chronic hypertension?	Martin	Conference abstract
52	Self-monitoring blood pressure in hypertensive pregnancies: the OPTIMUM-BP pilot randomised-controlled trial	Pealing	Conference abstract
53	OR21-4 Heart Health 4 Moms: A Randomized Trial to Reduce Cardiovascular Risk in Women with Recent Preeclampsia	Seely	Conference abstract
54	The Effects of High Dose Betamethasone Application on HELLP Syndrome	Caliskan	Full text not in English
55	The effect of nimodipine on retinal blood flow in pregnancy induced hypertension	Li	Full text not in English
56	Effect and mechanism of L-arginine therapy for fetal growth retardation due to pregnancy-induced hypertension	Nan	Full text not in English

57	Cardiovascular Risk Reduction in Women Following Hypertensive Disorders of Pregnancy - a Prospective, Randomised, Controlled Interventional Study	Riemer	Full text not in English
58	Clinical observation on therapeutical effect of prepared rhubarb in treating pregnancy induced hypertension	Wang	Full text not in English
59	The effect of adding lasix to antihypertensive treatment in controlling postpartum hypertension in women with preeclampsia		Trial registration only
60	Postpartum Intravenous Dexamethasone for Severely Preeclamptic Patients Without Hemolysis, Elevated Liver Enzymes, Low Platelets (HELLP) Syndrome: A Randomized Trial	Barrilleaux	Unable to extract data
61	Ketanserin versus dihydralazine for the treatment of severe hypertension in early-onset preeclampsia: a double blind randomized controlled trial	Bijvank	Unable to extract data
62	Ketanserin versus dihydralazine in the management of severe early-onset preeclampsia: Maternal outcome	Bolte	Unable to extract data
63	Does labetalol influence the development of proteinuria in pregnancy hypertension? A randomised controlled study	Cruickshank	Unable to extract data
64	Pre-eclampsia and nasal CPAP: Part 2. Hypertension during pregnancy, chronic snoring, and early nasal CPAP intervention	Dalva Poyares	Unable to extract data
65	Efficacy of normodyne-magnesium sulfate combination treatment on pregnancy-induced hypertension, and its effect on VEGF and Flt-1 levels	Diao	Unable to extract data

66	Antihypertensive Drug Effects on Placental Flow Velocity,Waveforms in Pregnant Women with Severe Hypertension	Duggan	Unable to extract data
67	Impact of Combined Nifedipine and Aspirin Therapy on Hemodynamics in Patients with Early Eclampsia	Fan	Unable to extract data
68	A randomised placebo controlled trial of loop diuretics in moderate/severe pre-eclampsia, following delivery	Gornall	Unable to extract data
69	Maternal and fetal haemodynamics in hypertensive pregnancies during maternal treatment with intravenous hydralazine or labetalolMaternal and fetal haemodynamics in hypertensive pregnancies during maternal treatment with intravenous hydralazine or labetalol	Harper	Unable to extract data
70	Effect of High-Dose Intravenous Vitamin C on Postpartum Oxidative Stress in Severe Preeclampsia	Korenc	Unable to extract data
71	Efficacy of an enhanced recovery nursing plan as a rooming-in practice for women with preeclampsia post-cesarean section	Li	Unable to extract data
72	Postpartum corticosteroids: Accelerated recovery from the syndrome of hemolysis, elevated liver enzymes, and low platelets (HELLP)	Magann	Unable to extract data
73	Optimal timing of delivery for pregnant individuals with mild chronic hypertension	Metz	Unable to extract data
74	The effect of serotonergic blockade in postpartum preeclamptic patients	Montenegro	Unable to extract data

75	Effect of l-arginine on blood pressure in pregnancy-induced hypertension: A randomized placebo-controlled trial	Neri	Unable to extract data
76	CHronic hypERtension and L-citRulline studY (CHERRY): an Early-Phase Randomised Controlled Trial in Pregnancy	Ormesher	Unable to extract data
77	Effects of calcium supplementation on markers of preeclampsia: randomized clinical trial	Pitilin	Unable to extract data
78	Randomized Trial to Reduce Cardiovascular Risk in Women with Recent Preeclampsia	Rich-Edwards	Unable to extract data
79	A randomized, double-blind, hemodynamic evaluation of nifedipine and labetalol in preeclamptic hypertensive emergencies	Scardo	Unable to extract data
80	The effect of labetalol and nifedipine MR on blood pressure in women with chronic hypertension in pregnancy	Shawkat	Unable to extract data
81	A Comparison of Labetalol Plus Hospitalization Versus Hospitalization Alone in the Management of Preeclampsia Remote From Term	Sibai	Unable to extract data
82	A randomized prospective comparison of nifedipine and bed rest versus bed rest alone in the management of preeclampsia remote from term	Sibai	Unable to extract data
83	Furosemide in postpartum management of severe preeclampsia: A randomized controlled trial	Veena	Unable to extract data
84	A Placebo Controlled Trial of Metoprolol in the Treatment of Hypertension in Pregnancy	Wichman	Unable to extract data

85	Effects of postpartum corticosteroids in patients with HELLP syndrome	Yalcin	Unable to extract data
86	Effects of Nifedipine Tablets Combined With Magnesium Sulfate on Blood Coagulation Index, Oxidative Stress, NO and ET-1 Levels in Patients With Pregnancy Hypertension	Yu	Unable to extract data
87	Efficacy of aspirin combined with labetalol on gestational hypertension and effect on serum PAPP-A, APN and HMGB1	Zhang	Unable to extract data
88	Efficacy of magnesium sulfate combined with nifedipine for pregnancy-induced hypertension syndrome and its relation to glucose and lipid metabolism	Zhang	Unable to extract data
89	Effects of magnesium sulfate and labetalol combined therapy on blood pressure and pregnancy outcomes in early-onset severe preeclampsia	Zhu	Unable to extract data
90	Feasibility and effectiveness of a lifestyle intervention after complicated pregnancies to improve risk factors for future cardiometabolic disease	Berks	Wrong population
91	A randomized trial of tight vs. less tight control of mild essential and gestational hypertension in pregnancy	El Guindy	Wrong population
92	Pregnancy outcomes of expectant management of stable mild to moderate chronic hypertension as compared with planned delivery	Hamed	Wrong population
93	The Control of Hypertension In Pregnancy Study pilot trial	Magee	Wrong population
94	Less-Tight versus Tight Control of Hypertension in Pregnancy	Magee	Wrong population
95	Effects of Antihypertensive Therapy During Pregnancy on Postpartum Blood Pressure Control	Martin	Wrong population

96	Blood pressure patterns in pregnant patients on oral ketanserin	Steyn	Wrong population
97	NO donors on top of anti-hypertensive therapy reduces complications in chronic hypertensive pregnancies with hypodynamic circulation	Vasapollo	Wrong population
98	Labetalol Versus Nifedipine as Antihypertensive Treatment for Chronic Hypertension in Pregnancy	Webster	Wrong population
99	Treatment of hypertension in pregnancy with methyldopa: a randomized double blind study	Weitz	Wrong population
100	Metoprolol in the Treatment DF Mild to Moderate Hypertension in Pregnancy-Effects on the Mother	Wichman	Wrong population
101	Treatment of pregnancy-induced hypertension compared with labetalol, low dose aspirin and placebo	Xiang	Wrong population
102	Planned early delivery or expectant management for late preterm pre-eclampsia (PHOENIX): a randomised controlled trial	Chappell	Wrong study design
103	Management of non-severe pregnancy hypertension – A summary of the CHIPS Trial (Control of Hypertension in Pregnancy Study) research publications	Magee	Wrong study design
104	Aspirin reduces long-term stroke risk in women with prior hypertensive disorders of pregnancy	Miller	Wrong study design
105	Home blood pressure monitoring detects unrevealed hypertension in women with a history of preeclampsia: results of the BP-PRESELF study	Muijsers	Wrong study design

106	Effect of Nursing Intervention on Pregnancy Outcomes, Blood Pressure, Postpartum Self-efficacy, and Quality of Life in Patients with Preeclampsia: An Observational Study	Qian	Wrong study design
107	Nifedipine outperforms labetalol: A comparative analysis of hypertension management in black pregnancies	Smith	Wrong study design
108	Low dose acetyl salicylic acid in severe preeclampsia	Toppozada	Wrong study design
109	Comparison Between Labetalol And Methyldopa In The Treatement Of Pre-Eclampsia	Akhtar	Timepoint of follow up
110	Oral Labetalol in Comparison with Methyldopa in Treatment of Gestational Hypertension as a First Line Drug	Akhter	Timepoint of follow up
111	Effect Of Lifestyle Modification Guidelines On Maternal And Fetal Outcomes Among Pregnant Women With Mild Preeclampsia	Ali	Timepoint of follow up
112	Postpartum Preeclampsia Management With Furosemide: A Randomized Clinical Trial	Ascarelli	Timepoint of follow up
113	Changes In Fetal And Maternal Doppler Parameters Observed During Acute Severe Hypertension Treatment With Hydralazine Or Labetalol: A Randomized Controlled Trial	Baggio	Timepoint of follow up
114	The effect of magnesium sulfate on maternal retinal blood flow in preeclampsia: a randomised placebo-controlled study	Belfort	Timepoint of follow up
115	Effect of magnesium sulfate on maternal brain blood flow in preeclampsia: A randomized, placebo-controlled study	Belfort	Timepoint of follow up

116	Safety of a physical therapy protocol for women with preeclampsia: a randomized controlled feasibility trial	Bertagnolli	Timepoint of follow up
117	The haemodynamic effects of ketanserin versus dihydralazinein severe early-onset hypertension in pregnancy	Bolte	Timepoint of follow up
118	Music therapy improves pregnancy-induced hypertension treatment efficacy	Cao	Timepoint of follow up
119	The clinical effect of aspirin combined with low-molecular-weight heparin in the treatment of severe preeclampsia and the combination's effect on pregnancy outcomes	Chang	Timepoint of follow up
120	Self-Monitoring Of Blood Pressure In Women With Pregnancy Hypertension: The BUMP2 Multicentre Randomised Controlled Trial	Chappell	Timepoint of follow up
121	Effects of different physical exercise types on health outcomes of individuals with hypertensive disorders of pregnancy: a prospective randomized controlled clinical study	Chen	Timepoint of follow up
122	An Exploratory Analysis of the Utility of Adding Cardiorespiratory Biofeedback in the Standard Care of Pregnancy-Induced Hypertension	Cullins	Timepoint of follow up
123	Effect of aerobic exercise versus device-guided breathing on gestational hypertension	El-Bandrawy	Timepoint of follow up
124	Methyldopa vs no drug treatment in the management of mild preeclampsia	Elhassan	Timepoint of follow up
125	Management Of Pregnancy-Induced Hypertension With Pindolol - Comparative Study With Methyldopa	Ellenbogen	Timepoint of follow up
126	L-Arginine Supplementation in Patients with Gestational Hypertension: A Pilot Study	Facchinetti	Timepoint of follow up

127	Effects of magnesium sulfate combined with labetalol on inflammatory stress and pregnancy outcome of patients with gestational hypertension	Gu	Timepoint of follow up
128	The effect of calcium supplementation on blood pressure in non-pregnant women with previous pre-eclampsia: A randomized placebo-controlled study	Hofmeyr	Timepoint of follow up
129	Clonidine Hydrochloride—A Safe and Effective Antihypertensive Agent in Pregnancy	Horvath	Timepoint of follow up
130	A prospective, randomized trial comparing the efficacy of dexamethasone and betamethasone for the treatment of antepartum HELLP (hemolysis, elevated liver enzymes, and low platelet count) syndrome	Isler	Timepoint of follow up
131	Evaluation of calcium-antagonist (Nifedipine) in the treatment of pre-eclampsia	Ismail	Timepoint of follow up
132	Nicardipine versus metoprolol in the treatment of hypertension during pregnancy: A randomised comparative trial	Jannet	Timepoint of follow up
133	Role of Calcium Supplementation on Pregnancy Induced Hypertension Outcomes	Jasim	Timepoint of follow up
134	Instrumental Soothing Music Reduces the Blood Pressure among Preeclamptic Women	Jayan	Timepoint of follow up
135	A Clinical Trial of Oral Hydration Therapy for the Management of Very Preterm Nonsevere Preeclampsia	Kamat	Timepoint of follow up
136	Comparison of Hydralazine and Labetalol to lower severe hypertension in pregnancy	Khan	Timepoint of follow up
137	Calcium supplementation on normotensive and hypertensive pregnant women	Knight	Timepoint of follow up
138	Oral nifedipine therapy in the management of severe preeclampsia	Kwawukume	Timepoint of follow up

139	Preeclampsia: Haemostatic Status and the Short-Term Effects of Methyldopa and Isradipine Therapy	Kyi	Timepoint of follow up
140	Short-term metabolic effects of isradipine and metoprolol in pre-eclampsia	Laivuori	Timepoint of follow up
141	Blood rheology in pre-eclampsia and intrauterine growth retardation: effects of blood pressure reduction with labetalol	Lang	Timepoint of follow up
142	Analysis of the Application Effect of Magnesium Sulfate Combined with Psychological Intervention in The Treatment of Pregnant Women with Preeclampsia	Lin	Timepoint of follow up
143	The effect of sublingual nifedipine on uteroplacental blood flow in hypertensive pregnancy	Lindow	Timepoint of follow up
144	Effect of a small dose of aspirin on quantitative test of 24-h urinary protein in patients with hypertension in pregnancy	Liu	Timepoint of follow up
145	Effect of low-molecular-weight heparin calcium combined with magnesium sulfate and labetalol on coagulation, vascular endothelial function and pregnancy outcome in early-onset severe preeclampsia	Liu	Timepoint of follow up
146	Intravenous Volume Expansion Therapy in Pregnancy-Induced Hypertension: The Role of Vasoactive Hormones	Lowe	Timepoint of follow up
147	Isradipine, a New Calcium Antagonist: Effects on Maternal and Fetal Hemodynamics	Lunell	Timepoint of follow up
148	Therapeutic potential of Omega-3 polyunsaturated fatty acids on the immune function of patients with hypertensive disorder complicating pregnancy and preeclampsia	Ma	Timepoint of follow up

149	Effect of the drug combination of magnesium sulfate and phentolamine on homocysteine and C-reactive protein in the serum of patients with pregnancy-induced hypertension syndrome	Ma	Timepoint of follow up
150	A Comparative Trial of Labetalol and Hydralazine in the Acute Management of Severe Hypertension Complicating Pregnancy	Mabie	Timepoint of follow up
151	A Comparative Study Of Intravenous Isradipine And Dihydralazine In The Treatment Of Severe Hypertension Of Pregnancy In Black Patients	Maharaj	Timepoint of follow up
152	Comparison between methyldopa and combination of methyldopa and nifedipine in terms of mean change in blood pressure in pregnancy induced hypertension	Malik	Timepoint of follow up
153	Sublingual Isosorbide Dinitrate in the Acute Control of Hypertension in Patients with Severe Preeclampsia	Martinez	Timepoint of follow up
154	Randomized, Controlled Trial Of Hydralazine Versus Nifedipine In Preeclamptic Women With Acute Hypertension	Martins-Costa	Timepoint of follow up
155	Furosemide to lower antenatal severe hypertension: a randomized placebo-controlled trial	Maykin	Timepoint of follow up
156	Effects of Guided Imagery on Blood Pressure in Pregnant Women with Hypertension: A Pilot Randomized Controlled Trial	Moffatt	Timepoint of follow up
157	Randomised controlled trial of methyldopa and isradipine in preeclampsia —effects on uteroplacental and fetal hemodynamics	Montan	Timepoint of follow up

158	Attempts at Dietary Alteration of Prostaglandin Pathways in the Management of Pre-eclampsia	Moodley	Timepoint of follow up
159	Impedance cardiography assessed treatment of acute severe pregnancy hypertension: a randomized trial	Morris	Timepoint of follow up
160	Evaluation of intravenous infusion of labetalol versus magnesium sulfate on cerebral hemodynamics of preeclampsia patients with severe features using transcranial doppler	Mowafy	Timepoint of follow up
161	Comparison of intravenous labetalol and hydralazine for severe hypertension in pregnancy in Northeastern Nigeria: A randomized controlled trial	Muhammad	Timepoint of follow up
162	Antepartum Aspirin Administration Reduces Activin A and Cardiac Global Longitudinal Strain in Preeclamptic Women	Naseem	Timepoint of follow up
163	Comparison of Role of Hydralazine Vs Labetalol in Mean Reduction of Mean Arterial Pressure in patients with Severe Preeclampsia	Naz	Timepoint of follow up
164	l-Arginine supplementation in women with chronic hypertension: impact on blood pressure and maternal and neonatal complications	Neri	Timepoint of follow up
165	24-Hour Ambulatory Blood Pressure Monitoring: A Comparison Between Transdermal Glyceryl-Trinitrate And Oral Nifedipine	Neri	Timepoint of follow up
166	Omeprazole Administration in Preterm Preeclampsia: a Randomized Controlled Trial to Study Its Effect on sFlt-1 (Soluble Fms-Like Tyrosine Kinase-1), PlGF (Placental Growth Factor), and ET-1 (Endothelin-1)	Neuman	Timepoint of follow up

167	Comparison of the Efficacy of Labetalol and Nifedipine in Preeclampsia: A Prospective Interventional Study	Nimbark	Timepoint of follow up
168	Effects of dietary nitrate supplementation, from beetroot juice, on blood pressure in hypertensive pregnant women: A randomised, double-blind, placebo-controlled feasibility trial	Ormesher	Timepoint of follow up
169	The effects of betamethasone treatment on clinical and laboratory features of pregnant women with HELLP syndrome	Ozer	Timepoint of follow up
170	The “Preeclampsia and Hypertension Target Treatment” study: a multicenter prospective study to evaluate the effectiveness of the antihypertensive therapy based on maternal hemodynamic findings	Pasquo	Timepoint of follow up
171	A randomised controlled trial of blood pressure self-monitoring in the management of hypertensive pregnancy. OPTIMUM-BP: A feasibility trial	Pealing	Timepoint of follow up
172	Early blood pressure control improves pregnancy outcome in primigravid women with mild hypertension	Phippard	Timepoint of follow up
173	A randomised placebo controlled trial of labetalol in the treatment of mild to moderate pregnancy induced hypertension	Pickles	Timepoint of follow up
174	The effect of labetalol versus placebo on arterial pressure and forearm venous tone in women with pregnancy induced hypertension	Pickles	Timepoint of follow up
175	Comparison of Magnesium and Methyldopa for the Control of Blood Pressure in Pregnancies Complicated with Hypertension	Rudnicki	Timepoint of follow up

176	Effects of prolonged oral supplementation with L-arginine on blood pressure and nitric oxide synthesis in preeclampsia	Rytlewski	Timepoint of follow up
177	Calcium Supplementation in Mild Preeclampsia Remote From Term: A Randomized Double-Blind Clinical Trial	Sanchez-Ramos	Timepoint of follow up
178	Pregnancy Outcomes of Nifedipine Compared With Labetalol for Oral Treatment of Mild Chronic Hypertension	Sanusi	Timepoint of follow up
179	Low dose aspirin does not influence the clinical course of women with mild PIH	Schiff	Timepoint of follow up
180	Plasma volume expansion in the treatment of pre-eclampsia	Sehgal	Timepoint of follow up
181	Comparison of Dexamethasone versus Betamethasone for the management of females with HELLP syndrome	Shazad	Timepoint of follow up
182	A comparison of no medication versus methyldopa or labetalol in chronic hypertension during pregnancy	Sibai	Timepoint of follow up
183	Dietary supplementation with L-arginine or placebo in women with pre-eclampsia	Staff	Timepoint of follow up
184	Calcium channel blockade (isradipine) in treatment of hypertension in pregnancy: A randomized placebo-controlled study	Swensson	Timepoint of follow up
185	Calcium Dobesilate Lowers the Blood Pressure in Mild to Moderate Midtrimester Hypertension	Tamas	Timepoint of follow up
186	Intravenous labetalol versus oral nifedipine for acute hypertension in pregnancy: effects on cerebral perfusion pressure	Tolcher	Timepoint of follow up

187	Effects of magnesium sulphuricum combined with low molecular weight heparin sodium on the blood pressure, D-D and hs-CRP levels in patients with severe preeclampsia	Tong	Timepoint of follow up
188	A randomised placebo-controlled trial of prolonged prednisolone administration to patients with HELLP syndrome remote from term	Van Runnard Heimel	Timepoint of follow up
189	Ketanserin versus alpha-methyldopa in the treatment of hypertension during pregnancy: a preliminary report	Voto	Timepoint of follow up
190	The effect of acute and chronic antihypertensive therapy on maternal and fetoplacental Doppler velocimetry	Walker	Timepoint of follow up
191	Efficacy of combined medication of nifedipine and magnesium sulfate on gestational hypertension and the effect on PAPP-A, VEGF, NO, Hcy and vWF	Wang	Timepoint of follow up
192	Magnesium Sulfate in combination with Nifedipine in the treatment of Pregnancy-Induced Hypertension	Xiang	Timepoint of follow up
193	The impact of aspirin combined with labetalol on coagulation function and pregnancy outcomes in pre-eclamptic pregnant women	Zhang	Timepoint of follow up

SUPPLEMENTARY TABLE 3

Quality assessment							No of patients		
No of participants (studies)	Design	Risk of bias	Inconsistency	Indirectness	Imprecision	Other considerations	Intervention	Control	Quality
The effect of pharmaceutical and nutraceutical interventions on postpartum systolic blood pressure when initiated antenatally									
450 (3 studies)	RCT	No serious risk of bias	Considerable	Downgraded by one level for surrogate marker	Downgraded by one level.	None	224	226	Moderate⊕⊕⊕◯
The effect of pharmaceutical and nutraceutical interventions on postpartum diastolic blood pressure when initiated antenatally									
450 (3 studies)	RCT	No serious risk of bias	Considerable	Downgraded by one level for surrogate marker	Downgraded by one level.	None	224	226	Moderate⊕⊕⊕◯
The effect of pharmaceutical and nutraceutical interventions on postpartum systolic blood pressure when initiated postnatally									
592 (7 studies)	RCT	Serious limitations, downgrade by one level	Considerable	Downgraded by one level for surrogate marker	Downgraded by one level.	None	294	298	Low ⊕⊕◯◯
The effect of pharmaceutical and nutraceutical interventions on postpartum diastolic blood pressure when initiated postnatally									
592 (7 studies)	RCT	Serious limitations, downgrade by one level	Considerable	Downgraded by one level for surrogate marker	Downgraded by one level.	None	294	298	Low ⊕⊕◯◯
The effect of remote blood pressure management on postpartum systolic blood pressure when initiated postnatally									
282 (2 studies)	RCT	No serious risk of bias	Low	Downgraded by one level for	Downgraded by one level	None	145	137	Moderate⊕⊕⊕◯

				surrogate marker	due to sample size.				
The effect of remote blood pressure management on postpartum diastolic blood pressure when initiated postnatally									
282<br>(2 studies)	RCT	No serious risk of bias	Low	Downgraded by one level for surrogate marker	Downgraded by one level due to sample size.	None	145	137	Moderate⊕⊕⊕◯

The quality of evidence across studies evaluating the effects of pharmaceutical and nutraceutical therapy or remote blood pressure management on postpartum blood pressure was assessed using the GRADE (Grading of Recommendations, Assessment, Development and Evaluation) framework. This assessment considered the total sample size and number of studies contributing to each outcome, with all included studies being randomized controlled trials (RCTs**)**. The risk of bias was evaluated using the RoB 2.0 tool, as described above. One study demonstrated a methodological limitation due to the absence of blinding among treating physicians, investigators, and nursing staff. Inconsistency was assessed through the degree of heterogeneity between studies, with considerable inconsistency leading to downgrading of the evidence. The use of blood pressure as a surrogate marker for future cardiovascular disease risk contributed to downgrading for indirectness. Furthermore, wide confidence intervals for both systolic and diastolic blood pressure outcomes, particularly in studies assessing pharmaceutical and nutraceutical therapy initiated antenatally or postnatally, resulted in downgrading for imprecision. The evidence for remote blood pressure management was also downgraded due to the small sample size. Overall, the GRADE assessment indicated moderate-quality evidence supporting antenatal pharmaceutical and nutraceutical therapy and remote blood pressure management, and low-quality evidence supporting postnatal pharmaceutical and nutraceutical therapy.

SUPPLEMENTARY TABLE 4

Further details describing the study populations, including the definition of HDP type, and the interventions, including extended descriptions, dose, and route of administration.

Study Information	Population		Intervention					
	HDP Type	Definition of HDP	Intervention Type	Intervention Description	Dose	Route of Administration	Intervention Duration	Control
Adair 2009[25] USA Double blind RCT Hospital	Preeclampsia	ACOG	Pharmaceutical and Nutraceutical Therapy	Digoxin-binding Fab fragment immunoglobulin (DIF)	2.0 ng/mL and using the subject's weight at delivery (dose = 2 x subject weight in kg/100)	IV	Within 15 minutes of the clamping of the umbilical cord after delivery, administered as an IV bolus over 10 minutes	Placebo (saline)
Aderibigbe 2023[26] USA Open RCT Hospital	Gestational HTN or chronic HTN	Gestational HTN: not defined<br><br>Chronic HTN: BP ≥140/90 mmHg on two separate occasions before 20 weeks of gestation	Pharmaceutical and Nutraceutical Therapy	Tight control of BP, administering antihypertensive therapy (chosen by the treating obstetrical provider) if SBP ≥140 or if DBP ≥90 mmHg	NA	NA	Beginning immediately after delivery, ending not specified	Liberal control of BP, administering antihypertensive therapy (chosen by the treating obstetrical provider) if BP ≥150/90 mmHg
Arias-Hernandez 2020[27] Mexico Single blind RCT Hospital	Preeclampsia	Clinical Practice Guide of the Mexican Institute of Social Security (SBP ≥160 mmHg and DBP ≥110 mmHg)	Pharmaceutical and Nutraceutical Therapy	Diltiazem	60 mg every 8 hours	Oral	2 days, beginning within 1 day post-delivery	Nifedipine 10 mg
Bartal 2023[28] USA Open RCT Hospital	Gestational HTN or chronic HTN	Gestational HTN: BP ≥140/90 mmHg on two separate occasions at 20 weeks of gestation<br><br>Chronic HTN: BP ≥140/90 mmHg on two separate occasions or the use of blood pressure	Pharmaceutical and Nutraceutical Therapy	HCTZ/Lisinopril	12.5mg HCTZ with 10mg lisinopril	Oral	Beginning within 3 days post-delivery, until first postpartum visit 7-10 days post delivery	Nifedipine 30mg

		medications before 20 weeks of gestation						
Barton 1990[29] USA Double blind RCT Hospital	Severe preeclampsia	1. SBP >180 mmHg or DBP >120 mmHg on one occasion; or<br>2. SBP between 160 and 180 mmHg or DBP between 110 and 120 mmHg that persists more than 2 hours despite bed rest; or<br>3. SBP >140 mmHg or DBP >90 mmHg on two separate occasions more than 6 hours apart despite bed rest plus one of the following:<br>a. Proteinuria >5 gm in 24 hours or ≥3+ on a urine dipstick measured on two separate occasions at least 2 hours apart in a patient with no previous history of renal disease<br>b. Urine output <500 mL in 24 hours or <80 mL in any 4-hour period despite a 250 mL fluid challenge<br>c. Pulmonary oedema without evidence of fluid overload<br>d . Alanine aminotransferase >100 IU/L<br>e. Platelet count <75,000 cells/ mm3<br>f. Seizure with no prior history of a seizure disorder	Pharmaceutical and Nutraceutical Therapy	Nifedipine	10 mg every 4 hours	Oral	2 days, beginning immediately after delivery	Placebo
Blue 2018[30] USA Double blind RCT Hospital	Preeclampsia, chronic HTN, or HELLP syndrome	ACOG	Pharmaceutical and Nutraceutical Therapy	Ibuprofen	600 mg every 6 hours	Oral	Beginning within 6 hours post-delivery, until discharge (minimum of 3 days)	Acetaminophen 650 mg

Cairns 2018[31] UK Open RCT Home	Gestational HTN or preeclampsia	NICE	Remote Blood Pressure Management	Daily BP self-monitoring using a validated device (Microlife WatchBP Home)	NA	NA	6 months, beginning after discharge	Usual care (BP monitored by community midwife and antihypertensive medication adjusted by general practitioner)
Chappell 2022[32] UK Open RCT Home	Gestational HTN or chronic HTN	Gestational HTN: sustained SBP ≥140 mmHg and/or DBP ≥90 mmHg after 20 weeks of gestation<br><br>Chronic HTN: sustained SBP ≥140 mmHg and/or DBP ≥90 mmHg, present at booking or before 20 weeks gestation, or receiving antihypertensive treatment outside pregnancy or at time of referral	Remote Blood Pressure Management	Daily BP self-monitoring using a validated device (Microlife WatchBP Home)	NA	NA	Beginning at recruitment, until delivery	Usual care (BP measured by health care professionals at regular antenatal clinics)
Cluver 2021[33] South Africa Double blind RCT Hospital	Preeclampsia	ISSHP, proteinuria only	Pharmaceutical and Nutraceutical Therapy	Metformin	3 g in divided doses of 500 mg	Oral	Beginning at recruitment, until delivery	Placebo
Cursino 2025[34] Brazil Triple blind RCT Hospital	Preeclampsia	ACOG	Pharmaceutical and Nutraceutical Therapy	Furosemide	40 mg/day	Oral	5 days, beginning 1 day after delivery	Placebo
Dabaghi 2019[35] Iran Single blind RCT Hospital	Preeclampsia or HELLP syndrome	Both not defined	Pharmaceutical and Nutraceutical Therapy	Furosemide	20 mg/day	Oral	5 days, beginning immediately after delivery	No medication
Elhassan 2002[36] Sudan Open RCT Hospital	Preeclampsia	DBP between 90 and 109 mmHg in two separate occasions 6 hours apart and 2+ of albumin	Pharmaceutical and Nutraceutical Therapy	Methyldopa	750 mg/day	Oral	Beginning at recruitment, until delivery	No medication

Elzayyat 2013[37] Egypt Single blind RCT Hospital	Preeclampsia	ACOG	Pharmaceutical and Nutraceutical Therapy	Magnesium sulphate	4 g pre delivery followed by 1 g/hour for 1 day post delivery	IV	Beginning 30 minutes pre delivery, until 1 day post delivery	No medication
Hashemi 2016[38] Iran Triple blind RCT Hospital	Preeclampsia	The National High Blood Pressure Education Program (2000)	Pharmaceutical and Nutraceutical Therapy	Aspirin	80 mg/day	Oral	Beginning at recruitment, until 2 months post delivery	Placebo
Hauspurg 2024[58] USA Open RCT Home	Gestational HTN or preeclampsia	ACOG	Lifestyle	Daily BP self-monitoring using a validated device (iHealth, Wireless Blood Pressure Monitor BP5) with and without an electric scale (iHealth Lite Wireless Scale, model HS4), along with self-help website access	NA	NA	6 months, beginning within 6 months of delivery	Self-help website access
Hladunewich 2006[39] USA Double blind RCT Hospital	Preeclampsia	SBP >140 mmHg and DBP >90 mmHg with the presence of proteinuria	Pharmaceutical and Nutraceutical Therapy	L-Arginine	Orally 3.5 g every 6 hours or intravenously 10 g every 8 hours	Oral and IV	Beginning within 1 day before delivery or immediately after delivery (a minority received intervention within a median of 6-8 days before delivery), until 10 days post delivery	Placebo
Hutchesson 2020[40] Australia Single blind RCT Home	Preeclampsia	Not defined	Lifestyle	Access to a lifestyle behaviour intervention (Be Healthe for your Heart) delivered via the program website and weekly email newsletters	NA	NA	3 months	Sent an email with links to the National Heart Foundation of Australia website (www.heartfoundation.org.au)
Isler 2003[41] USA Double blind RCT Hospital	HELLP syndrome	Haemolysis demonstrated by lactate dehydrogenase (LDH) ≥600 IU/L, hepatic dysfunction demonstrated by an elevation of aspartate aminotransferase (AST) ≥40 IU/L, and	Pharmaceutical and Nutraceutical Therapy	Dexamethasone	10 mg every 12 hours	IV	Beginning at recruitment, until criteria for discontinuation of the medication were fulfilled	Betamethasone 12 mg/day

		thrombocytopenia with platelets ≤100 000 /µL						
Kitt 2021[42] UK Open RCT Home	Gestational HTN or preeclampsia	NICE	Remote Blood Pressure Management	Daily BP self-monitoring using a validated device (Microlife WatchBP Home)	NA	NA	6 months, beginning after discharge	Usual care (BP monitored by community midwife and antihypertensive medication adjusted by general practitioner)
Kitt 2023[43] UK Open RCT Home	Gestational HTN or preeclampsia	Gestational HTN: SBP >140mmHg or DBP >90mnHg on more than two separate occasions after 20 weeks of gestation<br><br>Preeclampsia: NICE	Remote Blood Pressure Management	Daily BP self-monitoring using a validated device (OMRON Evolv monitor) which guides physician-approved antihypertensive medication titration	NA	NA	Beginning within 1-6 days post-delivery, ending not specified	Usual care (titration of antihypertensive treatment determined at the discretion of supervising health care professionals)
Kitt 2024[44] UK Open RCT Home	Gestational HTN or preeclampsia	Gestational HTN: SBP >140mmHg or DBP >90mnHg on more than two separate occasions after 20 weeks of gestation<br><br>Preeclampsia: NICE	Remote Blood Pressure Management	Daily BP self-monitoring using a validated device (OMRON Evolv monitor) which guides physician-approved antihypertensive medication titration	NA	NA	Beginning within 1-6 days post-delivery, ending not specified	Usual care (titration of antihypertensive treatment determined at the discretion of supervising health care professionals)
Movaghar 2024[45] Iran Triple blind RCT Hospital/Home	Preeclampsia	SBP between 140 and <160 mmHg or DBP between 90 to <110 mmHg, and the presence of protein in the urine	Pharmaceutical and Nutraceutical Therapy	Synbiotic capsule containing high amounts of probiotics (lactobacilli, bifidobacterial, and streptococci), along with fructo-oligosaccharide prebiotics	One capsule/day	Oral	Beginning at recruitment, until delivery	Placebo
Noronha 2017[46] Brazil Triple blind RCT Hospital	Gestational HTN or preeclampsia	The National High Blood Pressure Education Program (2000)	Pharmaceutical and Nutraceutical Therapy	Clonidine	0.1 mg at onset of a very high blood pressure episode	Oral	4 days, beginning at recruitment post delivery	Captopril 25 mg
Ormesher 2020[47] UK Double blind	Preeclampsia	ISSHP	Pharmaceutical and Nutraceutical Therapy	Enalapril	5 mg/day for 1 week, then 10 mg/day for 2 weeks, then 20 mg/day maintenance dose	Oral	6 months, beginning within 3 days post delivery	Placebo

RCT<br>Hospital								
Paidas<br>2020[48]<br>USA<br>Double blind<br>RCT<br>Hospital	Preeclampsia and superimposed preeclampsia	Preeclampsia:<br>• Gestational hypertension defined as a recorded SBP of ≥140 mm Hg or DBP of ≥90 mm Hg on 2 occasions at least 4 h apart (since the commencement of medical intervention in any facility)<br>OR<br>• Severe gestational hypertension defined as SBP of ≥160 mm Hg or DBP of ≥110 mm Hg, confirmed with a second assessment within a short interval (min)<br>AND new onset of any of the following:<br>• Proteinuria defined as ≥0.3 g protein per 24 h in a 12–24 h urine collection or PCR of ≥0.3 mg/mg (on a random sample or any collection period)<br>• Platelet count of <100,000/μL<br>• Serum creatinine concentrations of >1.1 mg/dL in the absence of other renal diseases<br>• Elevated liver transaminases to ≥twice the upper limit of normal<br>• Cerebral or visual symptoms<br>Superimposed preeclampsia:<br>• The start of antihypertensive	Pharmaceutical and Nutraceutical Therapy	Recombinant antithrombin therapy	250 mg loading dose followed by 2 g/day	IV	Beginning at recruitment, until delivery	Placebo

		medication, increasing the dose of a currently administered antihypertensive medication or adding a second antihypertensive medication after 20 wk of pregnancy for SBP of ≥160 mm Hg or DBP of ≥105 mm Hg in a subject who had a history of controlled hypertension before 20 wk of pregnancy<br>AND new onset of any of the following:<br>• Proteinuria defined as ≥0.3 g protein per 24 h in a 12–24 h urine collection or PCR ratio of ≥0.3 mg/mg (on a random sample or any collection period)<br>• Platelet count of <100,000/µL<br>• Serum creatinine concentrations of >1.1 mg/dL in the absence of other renal diseases<br>• Elevated liver transaminases to ≥twice the upper limit of normal<br>• Cerebral or visual symptoms						
Perdigao 2021[49]<br>USA<br>Double blind RCT<br>Hospital	Preeclampsia and superimposed preeclampsia	ACOG	Pharmaceutical and Nutraceutical Therapy	Furosemide	20 mg/day	Oral	5 days, beginning within 1 day post delivery	Placebo
Pihelgas 2024[50]<br>Canada	Preeclampsia	Not defined	Lifestyle	Referred to postpartum preeclampsia clinic	NA	NA	NA	Missed or declined referral to postpartum

C<br>Home								preeclampsia clinic
Siamansoori 2020[51]<br>Iran<br>Double blind RCT<br>Hospital	Preeclampsia	Not defined	Pharmaceutical and Nutraceutical Therapy	Furosemide	40 mg/day	Oral	Not defined	Methyldopa 750 mg/day
Suganya 2024[52]<br>India<br>Single blind RCT<br>Hospital	Preeclampsia	Not defined	Pharmaceutical and Nutraceutical Therapy	Furosemide & labetalol	40 mg/day of furosemide, labetalol dose not specified	Oral	5 days, beginning immediately after delivery	Labetalol, dose not specified
Trapani 2016[53]<br>Brazil<br>Double blind RCT<br>Hospital	Preeclampsia	SBP ≥140 mmHg or DBP ≥90 mmHg on two separate occasions after 20 weeks of gestation, along with associated proteinuria (>300 mg in 24 hours)	Pharmaceutical and Nutraceutical Therapy	Sildenafil citrate	150 mg/day	Oral	Beginning at recruitment, until delivery	Placebo
Vigil-De Gracia 1997[54]<br>Mexico<br>RCT<br>Hospital	HELLP syndrome	Clinical signs and symptoms consistent with a diagnosis of preeclampsia in addition to laboratory evidence of haemolysis, elevated liver enzymes, and thrombocytopenia	Pharmaceutical and Nutraceutical Therapy	Dexamethasone	10 mg immediately after delivery, then 10 mg at 12 hours post-delivery, then 10 mg at 1 day post delivery	IV	1 day, beginning immediately after delivery	No medication
Viteri 2018[55]<br>USA<br>Double blind RCT<br>Hospital/Home	Preeclampsia	SBP ≥140 mmHg or DBP ≥90 mmHg on two separate occasions from 20 weeks of gestation, along with associated proteinuria (spot urine/protein creatinine ratio ≥0.3 mg/dL)<br><br>Conversely, in the absence of proteinuria, a diagnosis of preeclampsia with severe features was made in the setting of either new-onset hypertension as defined or severe hypertension (sustained	Pharmaceutical and Nutraceutical Therapy	Torsemide	20 mg/day	Oral	5 days, beginning within 1 day post delivery	Placebo

		BP greater than or equal to 160 mm Hg systolic or 110 mm Hg diastolic) and new-onset of any of the following: thrombocytopenia (less than 100,000/ microliter), renal insufficiency (serum creatinine concentrations greater than 1.1 mg/dL or a doubling of serum creatinine concentration in the absence of other renal disease), impaired liver function (as evidenced by elevation of liver transaminases more or equal to twice normal upper limit concentration), pulmonary oedema, or cerebral or visual symptoms						
Wang 2017[57] China Double blind RCT Hospital	Preeclampsia	ACOG	Pharmaceutical and Nutraceutical Therapy	Nifedipine	10 mg, up to 5 doses until target BP reached	Oral	4 hours, beginning at recruitment (until target BP reached)	Labetalol in escalating doses of 20, 40, 80, 80, and 80 mg every 20 minutes until target BP reached
Wang 2024[56] USA Open RCT Home	Gestational HTN or preeclampsia	ACOG	Lifestyle	Utilisation of a neonatal sleep aid (SNOO responsive bassinet)	NA	NA	4 months, beginning after discharge	Usual care (safe sleep education)

Abbreviations: ACOG – American College of Obstetricians and Gynecologists; BP – blood pressure; DBP – diastolic blood pressure; HCTZ – hydrochlorothiazide; HDP – hypertensive disorder of pregnancy; HELLP - haemolysis, elevated liver enzymes and low platelets; HTN – hypertension; ISSHP – International Society for the Study of Hypertension in Pregnancy; IV – intravenous; NICE – National Institute for Health and Care Excellence; RCT – randomised control trial; SBP – systolic blood pressure

SUPPLEMENTARY TABLE 5

Recommendations for future research in women with hypertensive disorders of pregnancy.

Recommendations	Details
Trial Participants	- Accurate identification of high-risk women.<br>- Include racially, ethnically, geographically, and socially diverse populations.<br>- Distinguish women who develop OR are at high risk of:<br>• Preterm preeclampsia<br>• Preeclampsia<br>• Superimposed preeclampsia
Timing of Intervention	- Clarify timing differences between:<br>• Intervention trials<br>• Primary prevention trials<br>• 1st, 2nd and 3rd trimester vs postpartum
Potential Interventions to Be Tested	- Lifestyle<br>- Nutrition and physical activity for obesity prevention<br>- Nutrition-specific interventions including sodium reduction<br>- Pharmacological strategies including LDL-C lowering<br>- Remote blood pressure management<br>-Digital health technologies including wearables and sensors
Optimal Trial Endpoints	- Clinical endpoints:<br>• Stroke, MI, heart failure, ACS, cardiovascular death<br>• Blood pressure<br>• BMI<br>• Imaging-based endpoints:<br>- LV mass via MRI or echocardiography<br>• Serum biomarkers:<br>- Lipids, inflammatory markers<br>• Pulse wave velocity and augmentation index
Follow up	Longitudinal follow up beyond the immediate postpartum period

SUPPLEMENTARY FIGURE 1

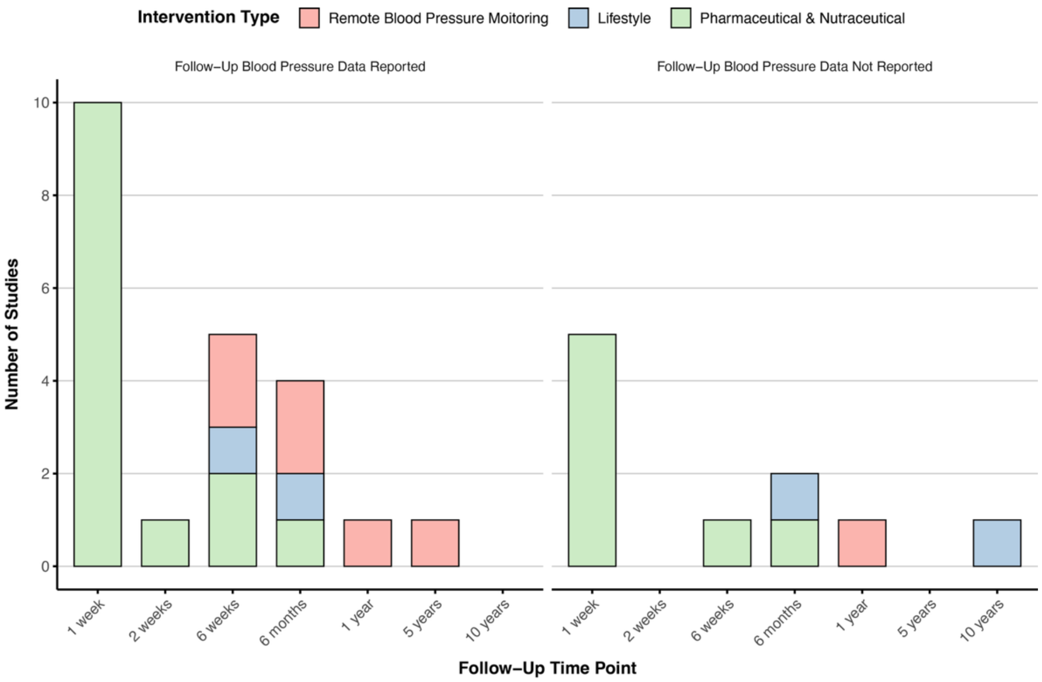

Supplementary Figure 1. Stacked bar plots show the number of studies reporting outcomes at each follow-up time point, stratified by intervention type. Intervention types include remote blood pressure management, lifestyle, and pharmaceutical and nutraceutical therapy. Studies are grouped by whether blood pressure data were reported as an outcome.

SUPPLEMENTARY FIGURE 2

A Leave-One-Out Analysis for SBP in Antenatal and Pharmaceutical Groups within 1-week postpartum

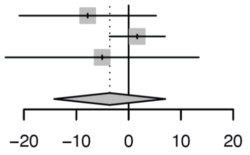

Omitted study	MD	95%–CI	P–value	Tau2	Tau	I2
Omitting Cluver, 2021 (Metformin, Day of Delivery)	−7.81	[−20.92; 5.29]	0.2424	76.1326	8.7254	84.9%
Omitting Movaghar, 2024 (Synbiotic, Day of Delivery)	1.66	[−3.67; 6.99]	0.5423	8.9446	2.9907	59.1%
Omitting Wang, 2017 (Nifedipine, Day of Delivery)	−5.06	[−23.55; 13.44]	0.5921	167.1427	12.9284	93.7%
**Random effects model**	**−3.60**	**[−14.25; 7.05]**	**0.5077**	**78.6169**	**8.8666**	**87.7%**

B Leave-One-Out Analysis for DBP in Antenatal and Pharmaceutical Groups within 1-week postpartum

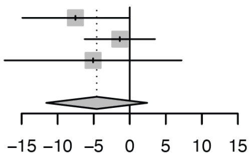

Omitted study	MD	95%–CI	P–value	Tau2	Tau	I2
Omitting Cluver, 2021 (Metformin, Day of Delivery)	−7.54	[−14.93; −0.14]	0.0457	22.8695	4.7822	80.0%
Omitting Movaghar, 2024 (Synbiotic, Day of Delivery)	−1.38	[−6.27; 3.52]	0.5813	9.2518	3.0417	74.0%
Omitting Wang, 2017 (Nifedipine, Day of Delivery)	−5.08	[−17.39; 7.22]	0.4180	73.7837	8.5897	93.5%
**Random effects model**	**−4.58**	**[−11.60; 2.44]**	**0.2006**	**33.9070**	**5.8230**	**87.5%**

C Leave-One-Out Analysis for SBP in Postnatal and Pharmaceutical Groups within 1-week postpartum

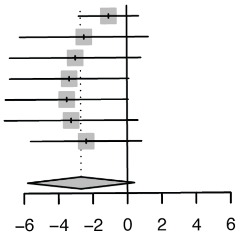

Omitted study	MD	95%–CI	P–value	Tau2	Tau	I2
Omitting Arias–Hernandez, 2020 (Nifedipine, 2 days)	−1.05	[−2.80; 0.69]	0.2374	1.4301	1.1959	34.0%
Omitting Cursino, 2025 (Furosemide, 5 days)	−2.49	[−6.23; 1.25]	0.1924	15.3466	3.9175	70.5%
Omitting Dabaghi, 2019 (Furosemide, 3 days)	−3.00	[−6.81; 0.82]	0.1233	16.4444	4.0552	74.9%
Omitting Noronha, 2017 (Clonidine, 4 days)	−3.36	[−6.82; 0.11]	0.0574	13.1802	3.6305	73.7%
Omitting Siamansoori, 2020 (Furosemide, 7 days)	−3.50	[−7.07; 0.07]	0.0544	12.9358	3.5966	67.3%
Omitting Suganya, 2024 (Furosemide + Labetalol, 5 days)	−3.25	[−7.14; 0.63]	0.1005	16.3325	4.0414	74.7%
Omitting Vigil–De Gracia, 1997 (Dexamethasone, 3 days)	−2.39	[−5.62; 0.84]	0.1472	12.1222	3.4817	73.1%
**Random effects model**	**−2.69**	**[−5.80; 0.41]**	**0.0893**	**11.8583**	**3.4436**	**70.0%**

D Leave-One-Out Analysis for DBP in Postnatal and Pharmaceutical Groups within 1-week postpartum

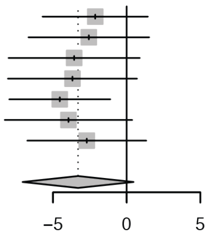

Omitted study	MD	95%–CI	P–value	Tau2	Tau	I2
Omitting Arias–Hernandez, 2020 (Nifedipine, 2 days)	−2.13	[−5.71; 1.45]	0.2425	17.1404	4.1401	94.4%
Omitting Cursino, 2025 (Furosemide, 5 days)	−2.57	[−6.67; 1.53]	0.2194	22.8791	4.7832	93.6%
Omitting Dabaghi, 2019 (Furosemide, 3 days)	−3.56	[−8.02; 0.89]	0.1170	27.6494	5.2583	95.7%
Omitting Noronha, 2017 (Clonidine, 4 days)	−3.68	[−8.07; 0.71]	0.1004	26.9640	5.1927	95.8%
Omitting Siamansoori, 2020 (Furosemide, 7 days)	−4.54	[−7.97; −1.11]	0.0096	14.7313	3.8381	86.0%
Omitting Suganya, 2024 (Furosemide + Labetalol, 5 days)	−3.95	[−8.28; 0.38]	0.0736	25.5783	5.0575	95.7%
Omitting Vigil–De Gracia, 1997 (Dexamethasone, 3 days)	−2.71	[−6.75; 1.34]	0.1897	23.2908	4.8261	95.6%
**Random effects model**	**−3.30**	**[−7.07; 0.47]**	**0.0865**	**22.7233**	**4.7669**	**95.1%**

Supplementary Figure 2. Leave-One-Out Sensitivity Analyses for Blood Pressure Outcomes Across Groups.

Panels A–D present leave-one-out sensitivity analyses conducted within random effects meta-analyses to assess the robustness of pooled estimates for systolic blood pressure (SBP) and diastolic blood pressure (DBP). Each forest plot displays the effect of omitting one study at a time on the overall mean difference (MD), along with the corresponding 95% confidence intervals (CI), p-values, and heterogeneity statistics (Tau^2, Tau, and I^2). These leave-one-out forest plots visually demonstrate the influence of each individual study on the overall pooled effect and provide insights into the consistency and heterogeneity of the findings across different intervention settings and time points.

SUPPLEMENTARY FIGURE 3

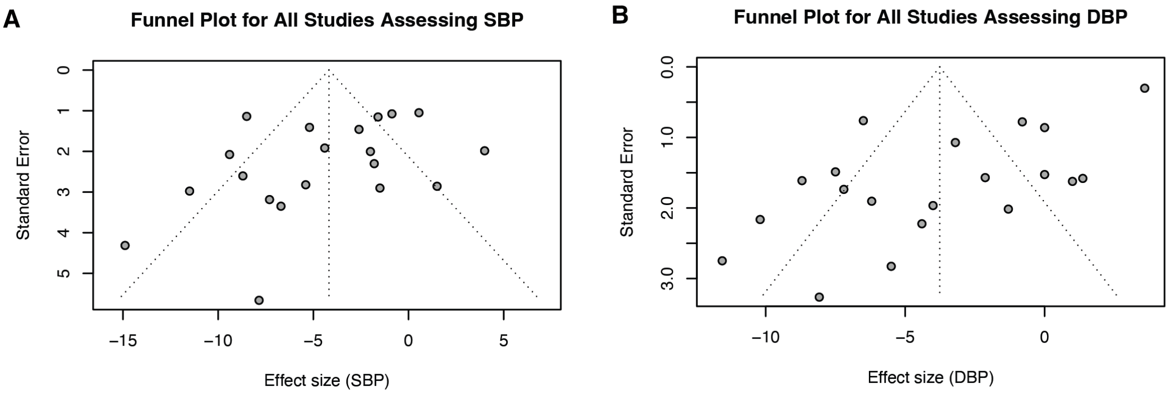

Supplementary Figure 3. Funnel plots assessing publication bias in meta-analyses of systolic (SBP) and diastolic blood pressure (DBP) outcomes.

Panels A–B present funnel plot analyses conducted within random effects meta-analyses to evaluate potential small-study effects and publication bias. Panel A displays the funnel plot for all studies reporting SBP, while Panel B shows the funnel plot for all studies reporting DBP.
